# Challenges in structural variant calling in low-complexity regions

**DOI:** 10.1093/gigascience/giaf154

**Published:** 2025-12-12

**Authors:** Qian Qin, Heng Li

**Affiliations:** Division of Rheumatology, Inflammation and Immunity, Brigham Women’s Hospital, Boston, MA 02115, USA; Department of Biomedical Informatics, Harvard Medical School, Boston, MA 02215, USA; Department of Data Science, Dana-Farber Cancer Institute, Boston, MA 02215, USA; Medical and Population Genetics Program, Broad Insitute of MIT and Harvard, Cambridge, MA 02142, USA

**Keywords:** structural variant, low-complexity regions, evaluation

## Abstract

**Background:**

Structural variants (SVs) are genomic differences $\ge$50 bp in length. They remain challenging to detect, even with long-sequence reads, and the sources of these difficulties are not well quantified.

**Results:**

We identified 35.4 Mb of low-complexity regions (LCRs) in GRCh38. Although these regions cover only 1.2% of the genome, they contain 69.1% of confident SVs in sample HG002. Across long-read SV callers, 77.3–91.3% of erroneous SV calls occur within LCRs, with error rates increasing with LCR length.

**Conclusion:**

SVs are enriched and difficult to call in LCRs. Special care needs to be taken for calling and analyzing these variants.

## Introduction

Structural variants (SVs) are $\ge$50-bp genomic variants and may have functional impacts [1]. Recent work based on high-quality long-read assemblies suggests there are broadly 25,000–35,000 SVs per human individual [2, 3]. Constructed by the Genome-In-A-Bottle (GIAB) group, the latest SV benchmark HG002-Q100 v1.1 [4] contains 28,188 SVs in 2.76 Gb of confident regions, consistent with the recent counts. In contrast, published in 2020 [5], the older HG002-SV benchmark v0.6 only contains 9,705 SVs in 2.66 Gb. This seems to suggest $\sim$18,000 SVs would fall in $\sim$100-Mb regions if we assume the SV v0.6 regions are contained in Q100 v1.1. Is this the correct interpretation?

This article shows that the differences between the 2 versions of the GIAB SV benchmarks are primarily driven by low-complexity regions (LCRs) that harbor repeatedly occurring motifs. The older benchmark excluded many of LCRs because it was hard to call them correctly. Although SV caller developers have noticed the difficulties in calling SVs around LCRs [5–7], they have not systematically quantified the effect of LCRs in SV calling. There is not a consensus on the number of SVs in LCRs or the error rate of them. Here, we identified LCRs jointly from the reference genome and the assemblies from the Human Pangenome Reference Consortium (HPRC) [2] and evaluated their impact on SV calling with multiple callers.

## Data Description

We applied longdust [8] to GRCh38 and identified 115.4 Mb of LCRs on assembled chromosomes. We filtered about half of them that overlap with alpha and HSAT2/3 centromeric repeats found by dna-brnn [9]. In total, 34.4 Mb of LCRs were left when we selected LCRs of 50 bp or longer.

GRCh38 represents only 1 human genome. It may miss polymorphic LCRs present in other human samples but missing from GRCh38. To look for these LCRs, we ran longdust on all 462 assemblies from HPRC and used the results to annotate variant bubbles in the minigraph graph of these assemblies [10]. A variant bubble was marked as an LCR if (i) $\ge$70% of the sequences in the bubble were LCRs in the source assemblies, and (ii) the sequences in the bubble were not annotated as segmental duplications (SegDup) by HPRC. Note that if an LCR falls in a long polymorphic SegDup, most of the sequences in the corresponding bubble will be annotated as SegDup but not as LCR. This is why we put SegDup at a higher priority over LCRs during annotation. To focus on common variants, we dropped non-GRCh38 alleles supported by $<$5 assemblies. We ignored HG002 when counting alleles because we will use this sample for benchmarking later.

We merged the common polymorphic LCRs and GRCh38 LCRs and added 5 bp to both ends of each LCR because LCR boundaries may not be exact, and by convention, insertions are often placed right before exact tandem repeats. This resulted in a BED file with 111,067 records, covering 35.4 Mb of GRCh38. Of the 29,291 records that overlap with common polymorphic LCRs in the HPRC minigraph graph, 3,918 are not observed on GRCh38, and 16.2% of the LCRs are intersected with the SegDup annotation from the “genomicSuperDups” track of the UCSC Genome Browser [11]. We see the overlap because an LCR consisting of several copies of a long repeat unit could also be considered a SegDup.

LCRs are closely related to tandem repeats. Longdust identifies most tandem repeats of $\ge$50 bp with $\ge$4 exact copies of repeat units as LCRs [8], though it often misses tandem repeats with fewer copies and may report additional regions without clear tandem patterns. In total, 83.5% of our LCRs overlap with tandem repeats found by TRF [12] v4.10 (option: 2 7 7 80 10 50 500 -l12), and 92.0% overlap with TR Catalog [13] v1.2.1. This catalog covers 238 Mb of GRCh38, much larger than our regions.

We applied the same procedure to the T2T-CHM13 genome [14] and found 79.6 Mb of LCRs, doubling the length of LCRs in GRCh38. Most of the additional regions came from centromeric satellites that are not HSAT2/3 or alpha repeats. If we exclude all types of satellites [15], only 31.2 Mb is left. The remaining difference in size from GRCh38 LCRs is probably caused by satellite annotation.

## Data Analysis

To understand the effect of LCRs in long-read SV calling, we measured the accuracy of SV calls stratified by LCR. We called SVs with 11 callers and compared them to both the new HG002-Q100 v1.1 [4] and the old HG002-SV v0.6 [5] benchmarks to demonstrate the impact of LCR in SV calling.

### Investigating the GIAB truth SVs

Of the 29,131 SVs of $\ge$50 bp in length that are contained in the confident regions in the new HG002-Q100 v1.1 benchmark [4], 943 have “*” as alternate alleles. We manually inspected the read alignment around some of these SVs and believe they are all redundant. Removing them from the truth left us with 28,188 SVs. The truvari [16] evaluation tool also filters SVs with “*” alleles.

The older HG002-SV v0.6 benchmark [5] is available only in the GRCh37 coordinate. To evaluate the SV calling accuracy on this benchmark, we lifted its confident regions over to GRCh38 with UCSC’s liftover web service, which failed on 0.03% of intervals. We still took SVs from HG002-Q100 as the ground truth. There are 11,985 HG002-Q100 overlapping with the lifted HG002-SV confident regions, more than the 9,705 SVs from the older HG002-SV benchmark. The difference is caused by the allele resolution. Suppose both haplotypes in HG002 harbor a 6-kb insertion to the same location of the reference genome. The inserted sequences, however, differ by 1 single-nucleotide polymorphism between them. The newer HG002-Q100 benchmark would consider this event as 2 heterozygous insertions, but the older HG002-SV benchmark would merge the 2 insertion alleles and consider them as 1 homozygous insertion. As a result, we counted 7,362 insertions in HG002-Q100 v1.1 but only 5,444 in HG002-SV v0.6, a sharp reduction. At the same time, the allele resolution may also affect deletions. If there are overlapping deletions of similar lengths between the 2 haplotypes, HG002-Q100 will encode them as 2 independent deletions, but HG002-SV may merge them and thus reduce the total counts. Overall, constructed from long-read assemblies, HG002-Q100 is more precise and more accurate than HG002-SV.

### Calling SVs from long reads

We acquired PacBio High-Fidelity reads from HPRC [17], aligned them to the primary assembly of GRCh38 with minimap2 [18], and called SVs with cuteSV v2.1.1 [19], DeBreak v1.0.2 [20], Delly v1.3.3 [21], longcallD v0.0.5 [22], pbsv v2.11.0 [23], Sawfish v0.12.10 [24], Sniffles2 v2.6.3 [6], SVDSS v2.1.0 [25], and SVIM v2.0.0 [26]. We used kanpig v1.1.0 [13] for genotyping SVs called by SVDSS, as is suggested in the documentation. Sniffles2 may optionally take tandem repetitive regions as input, but using this option slightly reduced its overall accuracy, so we only evaluated its default setting.

We also tried specialized tandem repeat callers, including TRGT [27] and ATaRVa [28]. These tools may output both long reference and long alternate alleles for 1 variant. Truvari was unable to correctly evaluate such variants and greatly overestimated false positives. We thus did not include tandem repeat callers in this work.

### Most SVs are located in LCRs

**Figure 1:**
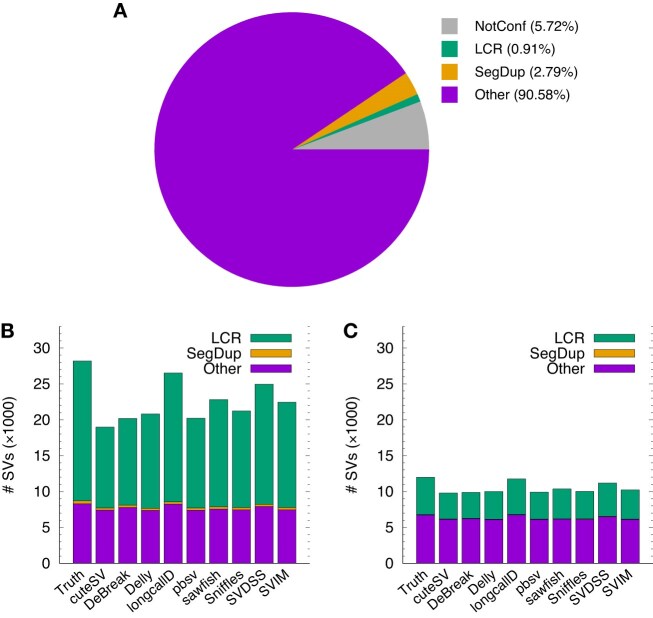
Number of HG002 structural variants (SVs) on GRCh38. (A) Lengths of regions. “NotConf” denotes not-confident regions in the HG002-Q100 v1.1 benchmark, excluding assembly gaps in GRCh38. A region classified to a previous type will not be counted toward the next type in the order of NotConf, LCR (low-complexity region), SegDup (segmental duplication), and Other. (B) Number of HG002-Q100 SVs stratified by LCR, SegDup, and the rest of the confident regions. An SV is classified as LCR (or SegDup) if $\ge$70% of its interval on GRCh38 overlaps with LCR (or SegDup). An SV classified as LCR will not be classified as SegDup. (C) Number of HG002-Q100 SVs in the HG002-SV v0.6 confident regions lifted over from GRCh37.

We stratified HG002 SVs by LCR and SegDup (Fig. 1). For an SV to be classified as LCR or SegDup, we required it to have large overlap with LCR or SegDup regions. Without this condition, a long deletion containing a short LCR would be falsely classified as LCR, which would inflate the number of LCR SVs. Across the SV callers, 59.4–67.7% of SV calls overlap with LCR, although LCR only accounts for 1.2% of GRCh38 or 0.9% of GIAB confident regions. SVs are highly enriched in LCR. In the ground truth, SVs in LCR contributed to 42.1% of total SV lengths. This suggests these SVs are shorter than the average.

Whereas the numbers of “Other” SVs in the HG002-Q100 confident regions are similar across callers, the numbers of LCR SVs differ greatly (Fig. 1B). SV callers that attempt to produce haplotype-resolved SVs, such as longcallD and SVDSS, call noticeably more SVs in LCR and SegDup. This trend is also observed in the older HG002-SV v0.6 confident regions (Fig. 1C). In the older HG002-SV regions, there are much fewer SVs in LCR and almost none in SegDup, although the numbers of SVs in Other regions are only reduced a little. This indicates that the main difference between HG002-Q100 and HG002-SV comes from LCRs.

### SVs in LCRs are harder to call correctly

We evaluated SV calls with truvari v5.3.0 [16], which performs multisequence alignment to normalize different variant representations and is recommended by GIAB. Having explored multiple truvari options, we settled on “bench --passonly --pick ac --dup-to-ins,” followed by “refine --use-original-vcfs,” as the resulting accuracy matched our manual inspection better.

**Figure 2:**
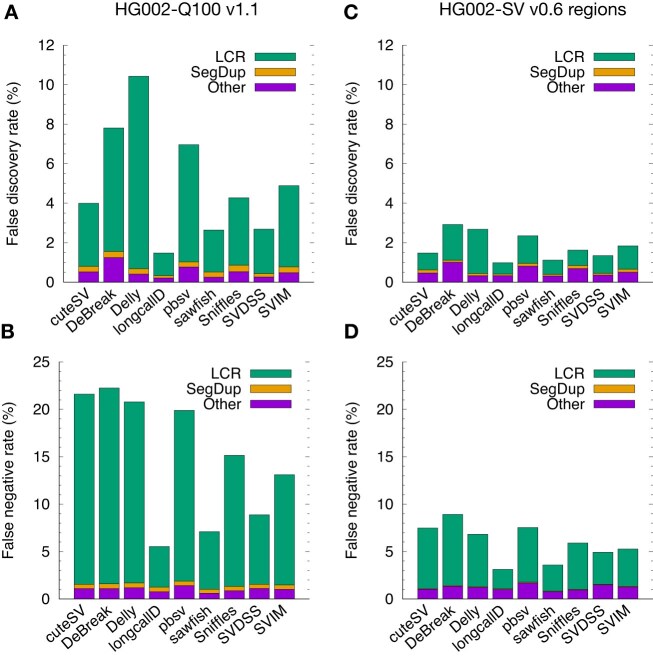
Accuracy of SV calls. (A) False discovery rate (FDR) of SVs in the HG002-Q100 confident regions, measured by truvari in the “refine” mode. SVs are stratified to LCR, SegDup, and Other in the same way as is described in Fig. 1. (B) False-negative rate (FNR) of SVs in HG002-Q100. (C) FDR in the HG002-SV confident regions. (D) FNR in HG002-SV.

On the new HG002-Q100 benchmark, 31.1–39.0% of SVs, depending on callers, are marked as “Other” (Fig. 1B), but only 5.2–14.0% of SV errors come from “Other” (Fig. 2A, B). This suggests SVs in the Other category are easier to call. In contrast, most errors, at 77.3–91.3%, are located in LCRs. SVs in SegDup are also difficult to call, but due to the small number of such SVs, they do not contribute much to the total number of errors. Furthermore, 66% of truth SVs are insertions. The error rates of insertions and of deletions are similar across callers, mostly within a few percent.

**Figure 3:**
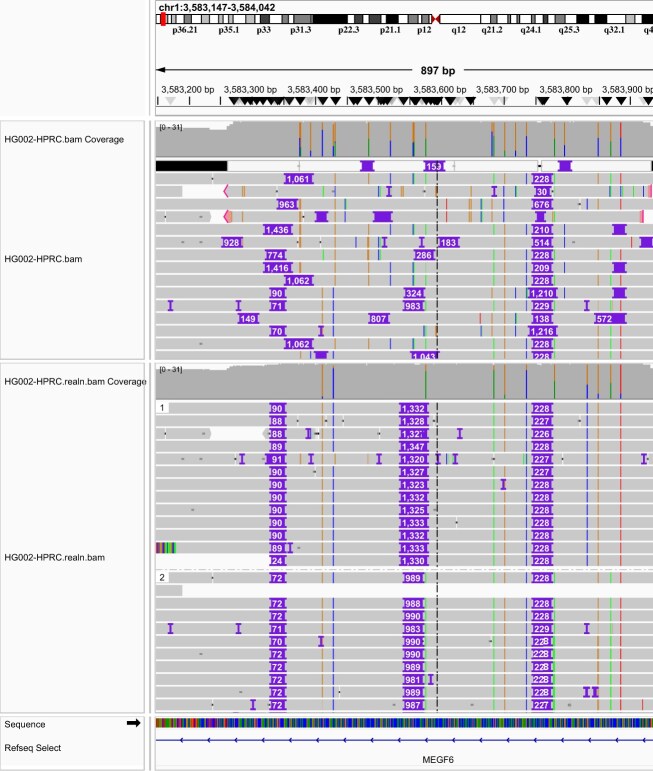
IGV screenshot of alignment around an LCR. The top panel shows the raw alignment by minimap2. The bottom panel shows the phased realignment by longcallD. There are 1,650 (= 90 + 1,332 + 228) inserted bases on the first haplotype in total and 1,290 (= 72 + 990 + 228) inserted bases on the second haplotype, identical to the HG002-Q100 ground truth.

Developed in our group but unpublished, longcallD achieves the lowest error rate (Fig. 2), mainly because it performs haplotype-aware multisequence realignment. As is shown in the top panel of Fig. 3, minimap2 often places gaps differently across reads and thus produces inconsistent alignment in long LCRs. This is primarily caused by the impure tandem repeat (TRF reports a 18-bp repeat unit of 91% averaged identity) where a small sequencing error may greatly alter gap placement. Minimap2 would not produce the desired alignment because it does not see other reads in the same region during pairwise alignment. Such inconsistency would confuse most SV callers. For this example, the SV callers in the order shown in Fig. 2, respectively, called +1,007/+1,007, +1,392/+1,392, +1,293/+1,293, +1,650/+1,290, +1,278/+1,278, +1,278/+1,668, +963/+1,191, +1,650/+1,290, and +1,353/+2,306 insertions on the 2 haplotypes. Only longcallD and SVDSS found the precise allele lengths of +1,650/+1,290. Nonetheless, truvari considered all callers correct. The error rate of most callers would probably be higher if we required precise allele matches.

**Figure 4:**
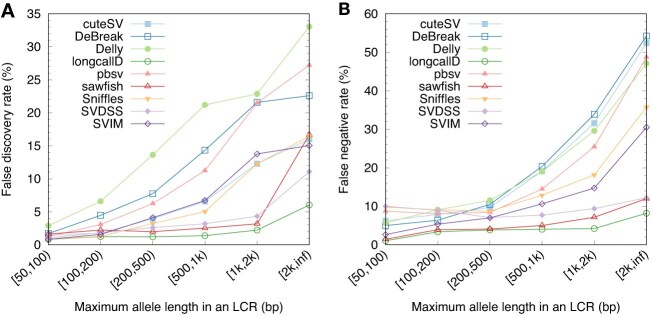
Accuracy of SV calls stratified by the maximum allele length in LCR. If an LCR is a common polymorphism (supported by $\ge$5 non-GRCh38 assemblies in HPRC), the maximum allele length equals the length of the longest allele aligned to the LCR; otherwise, the maximum allele length equals the length of the LCR on GRCh38.

We further stratified the errors by the maximum allele length of each LCR (Fig. 4) and observed increased error rates with maximum allele lengths. Some callers missed about half of SVs in $\ge$2-kb LCRs, even though HiFi reads are long enough to span most them. Simple algorithms without realignment or reassembly are not capable of calling SVs in long LCRs.

## Discussion

LCR SVs are a distinct class. Although LCRs contribute only to 1.2% of GRCh38, excluding alpha and HSAT2/3 repeats, they harbor more than half of long-read SV calls and an even higher fraction of SV calling errors. These errors are mainly caused by inconsistent read alignment, especially around long LCRs. Short-read SV calling may be affected more due to uncertainty in alignment around LCRs. On the other hand, we note that LCRs may overlap with coding exons of genes that have functional impacts [29], and they may also mediate gene expression [30, 31]. We would not want to filter all SVs overlapping LCRs.

For data analysts, we recommend stratifying SVs by LCR as LCR SVs are enriched with errors and result from different biological processes. For developers, we would like to emphasize the critical role of realignment or local reassembly in accurate SV calling. Most SVs in LCRs can still be called to decent accuracy with good algorithms.

Given accurate long reads at high coverage, we may also assemble the reads with haplotype-resolved assemblers [32, 33] and call variants from assembly-to-reference alignment [34]. Performing phasing and alignment within each haplotype, these assemblers are more powerful than most SV callers. As a matter of fact, the HG002-Q100 truth was derived this way.

We have analyzed only 1 human sample in this article. If mainstream SV callers are already struggling with long LCRs, merging their calls across different samples will be more problematic. When haplotype-resolved assembly is possible, calling variants across samples with pangenome-based methods [10, 35 ,36] will be the preferred approach, since by conducting multisequence alignment across samples, such methods can produce more consistent SV representations. They may also struggle with highly variable LCRs but will do better than traditional SV merging in most cases.

## Supplementary Material

giaf154_Authors_Response_To_Reviewer_Comments_Original_Submission

giaf154_Authors_Response_To_Reviewer_Comments_Revision_1

giaf154_GIGA-D-25-00409_original_submission

giaf154_GIGA-D-25-00409_Revision_1

giaf154_GIGA-D-25-00409_Revision_2

giaf154_Reviewer_1_Report_Original_Submission Zev Kronenberg, Ph.D. -- 10/21/2025

giaf154_Reviewer_1_Report_Revision_1 Zev Kronenberg, Ph.D. -- 12/4/2025

giaf154_Reviewer_3_Report_Revision_1 Shunichi Kosugi -- 12/2/2025

giaf154_Revision_2_Report_Original_Submission Wan-Ping Lee -- 10/31/2025

giaf154_Revision_3_Report_Original_Submission Shunichi Kosugi -- 11/6/2025

## Data Availability

Low-complexity regions (LCRs) are available at Zenodo [37] (files “chm13v2.lcr-v4.bed.gz” and “hg38.lcr-v4.bed.gz”). Scripts used for producing the LCRs and plots can be found at GitHub [38].

## Abbreviations

GIAB: Genome-In-A-Bottle group; HPRC: Human Pangenome Reference Consortium; kb: kilobase; LCR: low-complexity regions; Mb: megabase; SegDup: segmental duplication; SV: structural variant.
