# Supplementary material for: Challenges in structural variant calling in low-complexity regions
Source: Gigascience. 2025 Dec 12;14:giaf154. doi: 10.1093/gigascience/giaf154 (PMC12758381; doi:10.1093/gigascience/giaf154)
Supplement: giaf154_GIGA-D-25-00409_Revision_1 [file giaf154_giga-d-25-00409_revision_1.pdf]

GigaScience

Challenges in structural variant calling in low-complexity regions

--Manuscript Draft--

**Manuscript Number:**	GIGA-D-25-00409R1
**Full Title:**	Challenges in structural variant calling in low-complexity regions
**Article Type:**	Data Note
**Funding Information:**	National Human Genome Research Institute (R01HG010040) — Dr. Heng Li<br>National Human Genome Research Institute (R01HG014175) — Dr. Heng Li<br>National Human Genome Research Institute (U01HG013748) — Dr. Heng Li<br>National Human Genome Research Institute (U41HG010972) — Dr. Heng Li<br>National Cancer Institute (US) (U24CA294203) — Dr. Heng Li
**Abstract:**	Background: Structural variants (SVs) are genomic differences ≥50 bp in length. They remain challenging to detect even with long sequence reads, and the sources of these difficulties are not well quantified.<br>Results: We identified 35.4 Mb of low-complexity regions (LCRs) in GRCh38. Although these regions cover only 1.2% of the genome, they contain 69.1% of confident SVs in sample HG002. Across long-read SV callers, 77.3–91.3% of erroneous SV calls occur within LCRs, with error rates increasing with LCR length.<br>Conclusion: SVs are enriched and difficult to call in LCRs. Special care needs to be taken for calling and analyzing these variants.
**Corresponding Author:**	Heng Li<br>Dana-Farber Cancer Institute<br>Boston, UNITED STATES
**Corresponding Author Secondary Information:**	
**Corresponding Author's Institution:**	Dana-Farber Cancer Institute
**Corresponding Author's Secondary Institution:**	
**First Author:**	Qian Qin
**First Author Secondary Information:**	
**Order of Authors:**	Qian Qin<br>Heng Li
**Order of Authors Secondary Information:**	
**Response to Reviewers:**	Responses to reviewers' comments have been uploaded as a PDF file along with the manuscript.
**Additional Information:**	
**Question**	**Response**
Are you submitting this manuscript to a special series or article collection?	No
**Experimental design and statistics**	Yes

Powered by Editorial Manager® and ProduXion Manager® from Aries Systems Corporation

Full details of the experimental design and statistical methods used should be given in the Methods section, as detailed in our Minimum Standards Reporting Checklist. Information essential to interpreting the data presented should be made available in the figure legends.<br><br>Have you included all the information requested in your manuscript?	
**Resources**<br><br>A description of all resources used, including antibodies, cell lines, animals and software tools, with enough information to allow them to be uniquely identified, should be included in the Methods section. Authors are strongly encouraged to cite Research Resource Identifiers (RRIDs) for antibodies, model organisms and tools, where possible.<br><br>Have you included the information requested as detailed in our Minimum Standards Reporting Checklist?	Yes
**Availability of data and materials**<br><br>All datasets and code on which the conclusions of the paper rely must be either included in your submission or deposited in publicly available repositories (where available and ethically appropriate), referencing such data using a unique identifier in the references and in the "Availability of Data and Materials" section of your manuscript.<br><br>Have you have met the above requirement as detailed in our Minimum Standards Reporting Checklist?	Yes
GigaScience has policies and guidelines in place for the use of generative AI-	No

writing tools such as ChatGPT. If you have used such writing tools to assist with writing the manuscript this must be declared and cited in the text. Authors should not list AI-writing tools and other AI-assisted technologies as an author or co-author and should acknowledge that they are fully responsible for text generated or refined by AI-writing tools.<p><br><br>A summary of use (particularly in the introduction or among methods) needs to be included at the end of the paper, and the outputs should also be included as a supplementary file hosted in GigaDB or other open repositories. Please read our guidelines for more information. <p><br><br>By submitting to GigaScience, you are aware of the journal's AI-writing tools policy, and if you have declared use of such tools below, you have acknowledged this where appropriate in your manuscript and have made a summary of use and outputs available. <p><br><b>AI-assisted writing tools have been used in the preparation of this manuscript?	

Powered by Editorial Manager® and ProduXion Manager® from Aries Systems Corporation

LaTeX - Main Document (TeX file)

Click here to access/download;LaTeX - Main Document (TeX file);lcr-sv.tex

Placeholder for OUP logo oup.pdf

Placeholder for journal logo gigascience-logo.pdf

GigaScience, 2025, 1–5

doi: xx.xxxx/xxxx
Manuscript in Preparation
Data Note

DATA NOTE

Challenges in structural variant calling in low-complexity regions

Qian Qin[1] and Heng Li[2,3,4,*]

[1]Brigham Women's Hospital, 75 Francis St, Boston, MA 02115, USA and [2]Department of Biomedical Informatics, Harvard Medical School, 10 Shattuck St, Boston, MA 02215, USA and [3]Department of Data Science, Dana-Farber Cancer Institute, 450 Brookline Ave, Boston, MA 02215, USA and [4]Broad Insitute of MIT and Harvard, 415 Main St, Cambridge, MA 02142, USA

*hli@ds.dfci.harvard.edu

Abstract

Background: Structural variants (SVs) are genomic differences ≥50 bp in length. They remain challenging to detect even with long sequence reads, and the sources of these difficulties are not well quantified.

Results: We identified 35.4 Mb of low-complexity regions (LCRs) in GRCh38. Although these regions cover only 1.2% of the genome, they contain 69.1% of confident SVs in sample HG002. Across long-read SV callers, 77.3–91.3% of erroneous SV calls occur within LCRs, with error rates increasing with LCR length.

Conclusion: SVs are enriched and difficult to call in LCRs. Special care needs to be taken for calling and analyzing these variants.

Key words: structural variant; low-complexity regions; evaluation

Introduction

Structural variants (SVs) are ≥50bp genomic variants and may have functional impacts [1]. Recent work based on high-quality long-read assemblies suggests there are broadly 25,000–35,000 SVs per human individual [2, 3]. Constructed by the Genome-In-A-Bottle (GIAB) group, the latest SV benchmark HG002-Q100 v1.1 [4] contains 28,188 SVs in 2.76Gb of confident regions, consistent with the recent counts. In contrast, published in 2020 [5], the older HG002-SV benchmark v0.6 only contains 9,705 SVs in 2.66Gb. This seems to suggest ~18,000 SVs would fall in ~100Mb regions if we assume the SV v0.6 regions are contained in Q100 v1.1. Is this the correct interpretation?

This article shows that the differences between the two versions of the GIAB SV benchmarks are primarily driven by low-complexity regions (LCRs) that harbor repeatedly occurring motifs. The older benchmark excluded many of LCRs because it was hard to call them correctly. Although SV callers developers have noticed the difficulties in calling SVs around LCRs [5, 6, 7], they have not systematically quantified the effect of LCRs in SV calling. There is not a consensus on the number of SVs in LCRs or the error rate of them. Here, we identified LCRs jointly from the reference genome and the assemblies from the Human Pangenome Reference Consortium (HPRC) [2], and evaluated their impact on SV calling with multiple callers.

Data Description

We applied longdust [8] to GRCh38 and identified 115.4Mb of LCRs on assembled chromosomes. We filtered about half of them that overlap with alpha and HSAT2/3 centromeric repeats found by dna-brnn [9]. 34.4Mb of LCRs were left when we selected LCRs of 50bp or longer.

GRCh38 only represents one human genome. It may miss polymorphic LCRs present in other human samples but missing from GRCh38. To look for these LCRs, we ran longdust on all 462 assemblies from HPRC and used the results to annotate variant bubbles in the minigraph graph of these assemblies [10]. A variant bubble was marked as an LCR if (a) ≥70% of the sequences in the bubble were LCRs in the source assemblies, and (b) the sequences in the bubble were not annotated as segmental duplications (SegDup) by HPRC. Note that if an LCR falls in a long polymorphic SegDup, most of the sequences in the corresponding bubble will be annotated

Compiled on: November 19, 2025.
Draft manuscript prepared by the author.

as SegDup but not as LCR. This is why we put SegDup at a higher priority over LCRs during annotation. To focus on common variants, we dropped non-GRCh38 alleles supported by <5 assemblies. We ignored HG002 when counting alleles because we will use this sample for benchmarking later.

We merged the common polymorphic LCRs and GRCh38 LCRs and added 5bp to both ends of each LCR because LCR boundaries may not be exact and by convention, insertions are often placed right before exact tandem repeats. This resulted in a BED file with 111,067 records, covering 35.4Mb of GRCh38. 29,291 records overlap with common polymorphic LCRs in the HPRC minigraph graph. 3,918 of them are not observed on GRCh38. 16.2% of the LCRs are intersected with the SegDup annotation from the "genomicSuperDups" track of the UCSC Genome Browser [11]. We see the overlap because an LCR consisting of several copies of a long repeat unit could also be considered as a SegDup.

LCRs are closely related to tandem repeats. Longdust identifies most tandem repeats with ≥4 copies of repeat units as LCRs [10], but it often misses tandem repeats with fewer copies and may report additional regions without clear tandem patterns. 83.5% of our LCRs overlap with tandem repeats found by TRF [12] v4.10 (option `2 7 7 80 10 50 500 -l12`) and 92.0% overlap with TR Catalog [13] v1.2.1 which covers 238Mb of GRCh38, much larger than our regions.

We applied the same procedure to the T2T-CHM13 genome [14] and found 79.6Mb of LCRs, doubling the length of LCRs in GRCh38. Most of the additional regions came from centromeric satellites that are not HSAT2/3 or alpha repeats. If we exclude all types of satellites [15], only 31.2Mb is left. The remaining difference in size from GRCh38 LCRs is probably caused by satellite annotation.

Data Analysis

To understand the effect of LCRs in long-read SV calling, we measured the accuracy of SV calls stratified by LCR. We called SVs with 11 callers and compared them to both the new HG002-Q100 v1.1 [4] and the old HG002-SV v0.6 [5] benchmarks to demonstrate the impact of LCR in SV calling.

Investigating the GIAB truth SVs

There are 29,131 SVs of ≥50bp in length contained in the confident regions in the new HG002-Q100 v1.1 benchmark [4]. 943 of them have "*" as alternate alleles. We manually inspected the read alignment around some of these SVs and believe they are all redundant. Removing them from the truth left us with 28,188 SVs. The truvari [16] evaluation tool also filters SVs with "*" alleles.

The older HG002-SV v0.6 benchmark [5] is only available in the GRCh37 coordinate. To evaluate the SV calling accuracy on this benchmark, we lifted its confident regions over to GRCh38 with UCSC's liftover web service which failed on 0.03% of intervals. We still took SVs from HG002-Q100 as the ground truth. There are 11,985 HG002-Q100 overlapping with the lifted HG002-SV confident regions, more than the 9,705 SVs from the older HG002-SV benchmark. The difference is caused by the allele resolution. Suppose both haplotypes in HG002 harbor a 6kb insertion to the same location of the reference genome. The inserted sequences however differ by one SNP between them. The newer HG002-Q100 benchmark would consider this event as two heterozygous insertions, but the older HG002-SV benchmark would merge the two insertion alleles and consider them as one homozygous insertion. As a result, we counted 7,362 insertions in HG002-Q100 v1.1 but only 5,444 in HG002-SV v0.6, a sharp reduction. At the same time, the allele resolution may also affect deletions. If there are overlapping deletions of similar lengths between the two haplotypes, HG002-Q100 will encode them two independent deletions, but HG002-SV may merge them and thus reduce the total counts. Overall, constructed from long-read assemblies, HG002-Q100 is more precise and more accurate than HG002-SV.

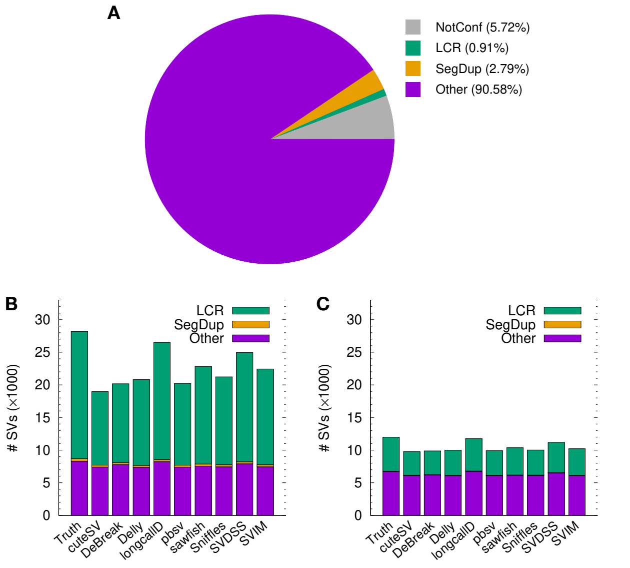

Figure 1. Number of HG002 structural variants (SVs) on GRCh38. (A) Lengths of regions. "NotConf" denotes not-confident regions in the HG002-Q100 v1.1 benchmark, excluding assembly gaps in GRCh38. A region classified to a previous type will not be counted towards the next type in the order of NotConf, LCR (low-complexity region), SegDup (segmental duplication) and Other. (B) Number of HG002-Q100 SVs stratified by LCR, SegDup and the rest of the confident regions. An SV is classified as LCR (or SegDup) if ≥70% of its interval on GRCh38 overlaps with LCR (or SegDup). An SV classified as LCR will not be classified as SegDup. (C) Number of HG002-Q100 SVs in the HG002-SV v0.6 confident regions lifted over from GRCh37.

Calling SVs from long reads

We acquired PacBio High-Fidelity reads from HPRC [17], aligned them to the primary assembly of GRCh38 with minimap2 [18] and called SVs with cuteSV v2.1.1 [19], DeBreak v1.0.2 [20], Delly v1.3.3 [21], longcallD v0.0.5 [22], pbsv v2.11.0 [23], Sawfish v0.12.10 [24], Sniffles2 v2.6.3 [6], SVDSS v2.1.0 [25], and SVIM v2.0.0 [26]. We used kanpig v1.1.0 [13] for genotyping SVs called by SVDSS as is suggested in the documentation. Sniffles2 may optionally take tandem repetitive regions as input, but using this option slightly reduced its overall accuracy, so we only evaluated its default setting.

We also tried specialized tandem repeat callers including TRGT [27] and ATaRVa [28]. These tools may output both long reference and long alternate alleles for one variant. Truvari was unable to correctly evaluate such variants and greatly overestimated false positives. We thus did not include tandem repeat callers in this work.

Most SVs are located in LCRs

We stratified HG002 SVs by LCR and SegDup (Fig. 1). For an SV to be classified as LCR or SegDup, we required it to have large overlap with LCR or SegDup regions. Without this condition, a long deletion containing a short LCR would be falsely classified as LCR, which would inflate the number of LCR SVs. Across the SV callers, 59.4–67.7% of SV calls overlap with LCR, although LCR only accounts for 1.2% of GRCh38 or 0.9% of GIAB confident regions. SVs are highly enriched in LCR. On the ground truth, SVs in LCR contributed to 42.1% of total SV lengths. This suggests these SVs are shorter than the average.

Whereas the numbers of "Other" SVs in the HG002-Q100 confident regions are similar across callers, the numbers of LCR SVs differ greatly (Fig. 1B). SV callers that attempt to produce haplotype-

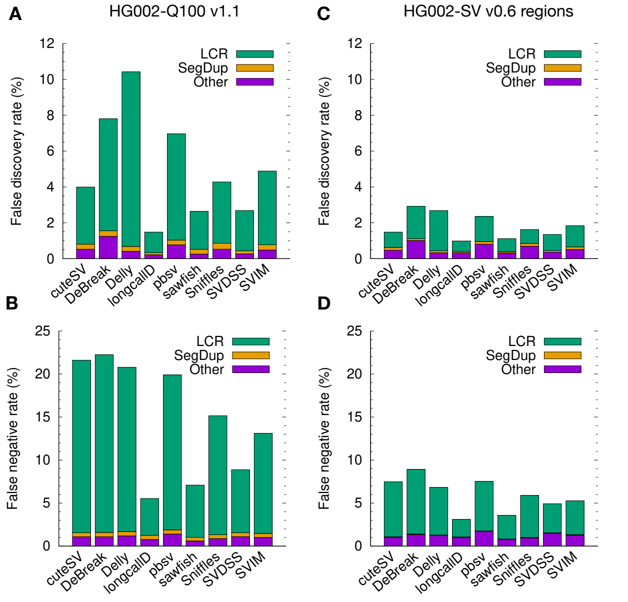

Figure 2. Accuracy of SV calls. (A) False discovery rate (FDR) of SVs in the HG002-Q100 confident regions, measured by truvari in the "refine" mode. SVs are stratified to LCR, SegDup and Other in the same way as is described in Fig. 1. (B) False negative rate (FNR) of SVs in HG002-Q100. (C) FDR in the HG002-SV confident regions. (D) FNR in HG002-SV.

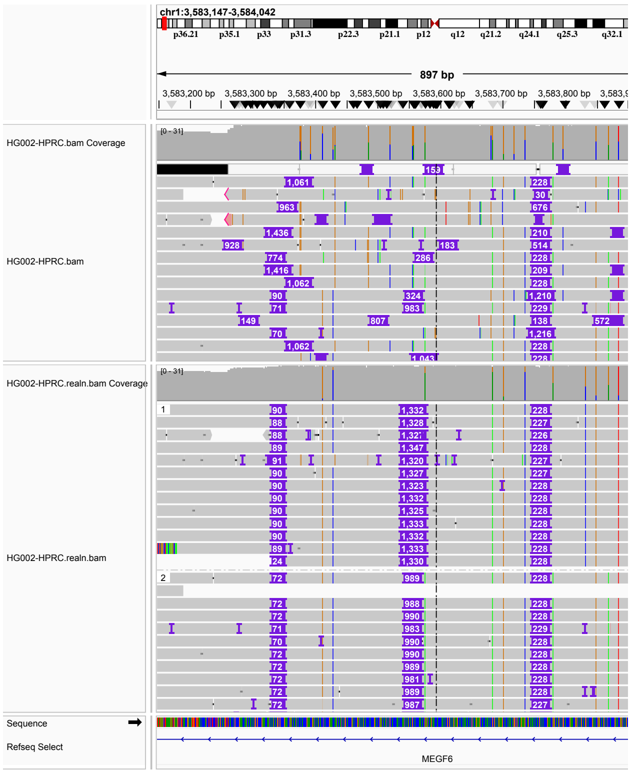

Figure 3. IGV screenshot of alignment around an LCR. The top panel shows the raw alignment by minimap2. The bottom panel shows the phased realignment by longcallD. There are 1650 (=90+1332+228) inserted bases on the first haplotype in total and 1290 (=72+990+228) inserted bases on the second haplotype, identical to the HG002-Q100 ground truth.

resolved SVs, such as longcallD and SVDSS, call noticeably more SVs in LCR and SegDup. This trend is also observed in the older HG002-SV v0.6 confident regions (Fig. 1C). In the older HG002-SV regions, there are much fewer SVs in LCR and almost none in SegDup, although the numbers of SVs in Other regions are only reduced a little. This indicates that the main difference between HG002-Q100 and HG002-SV comes from LCRs.

SVs in LCRs are harder to call correctly

We evaluated SV calls with truvari v5.3.0 [16], which performs multi-sequence alignment to normalize different variant representations and is recommended by GIAB. Having explored multiple truvari options, we settled on "`bench --passonly --pick ac --dup-to-ins`" followed by "`refine --use-original-vcfs`" as the resulting accuracy matched our manual inspection better.

On the new HG002-Q100 benchmark, 31.1–39.0% of SVs, depending on callers, are marked as "Other" (Fig. 1B), but only 5.2–14.0% of SV errors come from "Other" (Fig. 2A and 2B). This suggests SVs in the Other category are easier to call. In contrast, the majority of errors, at 77.3–91.3%, are located in LCRs. SVs in SegDup are also difficult to call, but due to the small number of such SVs, they do not contribute much to the total number of errors.

Developed in our group but unpublished, longcallD achieves the lowest error rate (Fig. 2) mainly because it performs haplotype-aware multi-sequence realignment. As is shown in the top panel of Fig. 3, minimap2 often places gaps differently across reads and thus produces inconsistent alignment in long LCRs. This happens because minimap2 does not see other reads in the same region during pairwise alignment. It is not apparent that there are only two haplotypes in this region. Such inconsistency would confuse most SV callers. For this example, the SV callers in the order shown in Fig. 2, respectively, called +1007/+1007, +1392/+1392, +1293/+1293, +1650/+1290, +1278/+1278, +1278/+1668, +963/+1191, +1650/+1290 and +1353/+2306 insertions on the two haplotypes. Only longcallD and SVDSS found the precise allele lengths of +1650/+1290. Nonetheless, truvari considered all callers correct. The error rate of most callers would probably be higher if we required precise allele matches.

We further stratified the errors by the maximum allele length of each LCR (Fig. 4) and observed increased error rates with maximum allele lengths. Some callers missed about half of SVs in ≥2kb LCRs, even though HiFi reads are long enough to span most them. Simple algorithms without realignment or reassembly are not capable of calling SVs in long LCRs.

Discussion

LCR SVs are a distinct class. Although LCRs only contribute to 1.2% of GRCh38 excluding alpha and HSAT2/3 repeats, they harbor more than half of long-read SV calls and an even higher fraction of SV calling errors. These errors are mainly caused by inconsistent read alignment especially around long LCRs. Short-read SV calling may be affected more due to uncertainty in alignment around LCRs.

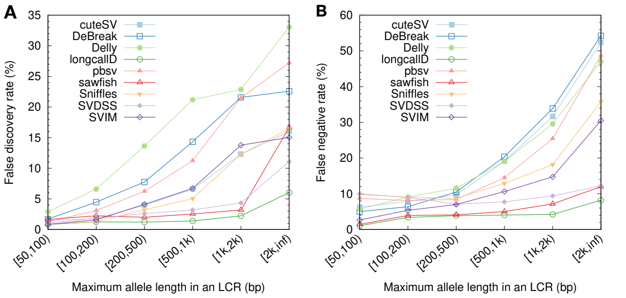

Figure 4. Accuracy of SV calls stratified by the maximum allele length in LCR. If an LCR is a common polymorphism (supported by ≥5 non-GRCh38 assemblies in HPRC), the maximum allele length equals to the length of the longest allele aligned to the LCR; otherwise, the maximum allele length equals to the length of the LCR on GRCh38.

On the other hand, we note that LCRs may overlap with coding exons of genes that have functional impacts [29] and they may also mediate gene expression [30, 31]. We would not want to filter all SVs overlapping LCRs.

For data analysts, we recommend stratifying SVs by LCR as LCR SVs are enriched with errors and are resulted from different biological processes. For developers, we would like to emphasize the critical role of realignment or local reassembly in accurate SV calling. Most SVs in LCRs can still be called to decent accuracy with good algorithms.

Given accurate long reads at high coverage, we may also assemble the reads with haplotype-resolved assemblers [32, 33] and call variants from assembly-to-reference alignment [34]. Performing phasing and alignment within each haplotype, these assemblers are more powerful than most SV callers. As a matter of fact, the HG002-Q100 truth was derived this way.

We have only analyzed one human sample in this article. If mainstream SV callers are already struggling with long LCRs, merging their calls across different samples will be more problematic. When haplotype-resolved assembly is possible, calling variants across samples with pangenome-based methods [10, 35, 36] will be the preferred approach as conducting multi-sequence alignment across samples, such methods can produce more consistent SV representations. They may also struggle with highly variable LCRs, but will do better than traditional SV merging in most cases.

Data Availability

LCRs are available at https://doi.org/10.5281/zenodo.10903864 (file "chm13v2.lcr-v4.bed.gz" and "hg38.lcr-v4.bed.gz"). Scripts used for producing the LCRs and plots can be found at https://github.com/lh3/lcr-sv.

Declarations

List of abbreviations

GIAB: Genome-In-A-Bottle group; HPRC: Human Pangenome Reference Consortium; kb: kilobase; LCR: low-complexity regions; Mb: megabase; SegDup: segmental duplication; SV: structural variant.

Competing Interests

The authors declare they have no competing interests.

Funding

This work is supported by National Institute of Health grant R01HG010040, R01HG014175, U24CA294203, U01HG013748 and U41HG010972 (to H.L.).

Author's Contributions

H.L. conceived the project. Q.Q. produced structural variant calls. Q.Q. and H.L. analyzed the data and drafted the manuscript.

Acknowledgements

We would like to acknowledge the National Genome Research Institute (NHGRI) for funding the following grants supporting the creation of the human pangenome reference: U41HG010972, U01HG010971, U01HG013760, U01HG013755, U01HG013748, U01HG013744, R01HG011274, and the Human Pangenome Reference Consortium (BioProject ID: PRJNA730823).

References

1. Eichler EE. Genetic Variation, Comparative Genomics, and the Diagnosis of Disease. N Engl J Med 2019;381:64–74.
2. Liao WW, Asri M, Ebler J, Doerr D, Haukness M, et al. A draft human pangenome reference. Nature 2023;617:312–324.
3. Logsdon GA, Ebert P, Audano PA, Loftus M, Porubsky D, et al. Complex genetic variation in nearly complete human genomes. Nature 2025;644:430–441.
4. Hansen NF, Dwarshuis N, Ji HJ, Rhie A, Loucks H, et al. A complete diploid human genome benchmark for personalized genomics. bioRxiv 2025;p. 2025.09.21.677443.
5. Zook JM, Hansen NF, Olson ND, Chapman L, Mullikin JC, et al. A robust benchmark for detection of germline large deletions and insertions. Nat Biotechnol 2020;38:1347–1355.
6. Smolka M, Paulin LF, Grochowski CM, Horner DW, Mahmoud M, et al. Detection of mosaic and population-level structural variants with Sniffles2. Nat Biotechnol 2024;42:1571–1580.
7. Keskus AG, Bryant A, Ahmad T, Yoo B, Aganezov S, et al. Severus detects somatic structural variation and complex rearrangements in cancer genomes using long-read sequencing. Nat Biotechnol 2025;.
8. Li H, Li B. Finding low-complexity DNA sequences with longdust. ArXiv 2025;p. arXiv:2509.07357v1.
9. Li H. Identifying centromeric satellites with dna-brnn. Bioinformatics 2019;35:4408–4410.
10. Li H, Feng X, Chu C. The design and construction of reference pangenome graphs with minigraph. Genome Biol 2020;21:265.
11. Perez G, Barber GP, Benet-Pages A, Casper J, Clawson H, et al. The UCSC Genome Browser database: 2025 update. Nucleic Acids Res 2025;53:D1243–D1249.
12. Benson G. Tandem repeats finder: a program to analyze DNA sequences. Nucleic Acids Res 1999;27:573–80.
13. English AC, Cunial F, Metcalf GA, Gibbs RA, Sedlazeck FJ. K-mer analysis of long-read alignment pileups for structural variant genotyping. Nat Commun 2025;16:3218.
14. Nurk S, Koren S, Rhie A, Rautiainen M, Bzikadze AV, et al. The complete sequence of a human genome. Science 2022;376:44–53.
15. Altemose N, Logsdon GA, Bzikadze AV, Sidhwani P, Langley SA, et al. Complete genomic and epigenetic maps of human centromeres. Science 2022;376:eabl4178.
16. English AC, Menon VK, Gibbs RA, Metcalf GA, Sedlazeck FJ. Truvari: refined structural variant comparison preserves allelic diversity. Genome Biol 2022;23:271.
17. Human Pangenome Reference Consortium;. https://human-pangenomics.s3.amazonaws.com/submissions/80d00e88-7a92-46d8-88c7-48f1486e11ed--HG002_PACBIO_REVIO/m84039_230117_233243_s1.hifi_reads.default.bam.
18. Li H. Minimap2: pairwise alignment for nucleotide sequences. Bioinformatics 2018;34:3094–3100.
19. Jiang T, Liu Y, Jiang Y, Li J, Gao Y, et al. Long-read-based human genomic structural variation detection with cuteSV. Genome Biol 2020;21:189.
20. Chen Y, Wang AY, Barkley CA, Zhang Y, Zhao X, et al. Deciphering the exact breakpoints of structural variations using long sequencing reads with DeBreak. Nat Commun 2023;14:283.
21. Rausch T, Zichner T, Schlattl A, Stütz AM, Benes V, Korbel JO. DELLY: structural variant discovery by integrated paired-end and split-read analysis. Bioinformatics 2012;28:i333–i339.
22. ;. https://github.com/yangao07/longcallD.
23. ;. https://github.com/PacificBiosciences/pbsv.
24. Saunders CT, Holt JM, Baker DN, Lake JA, Belyeu JR, et al. Sawfish: improving long-read structural variant discovery and genotyping with local haplotype modeling. Bioinformatics 2025;41:btaf136.
25. Denti L, Khorsand P, Bonizzoni P, Hormozdiari F, Chikhi R. SVDSS: structural variation discovery in hard-to-call genomic regions using sample-specific strings from accurate long reads.

Nat Methods 2023;20:550–558.
26. Heller D, Vingron M. SVIM: structural variant identification using mapped long reads. Bioinformatics 2019;35:2907–2915.
27. Dolzhenko E, English A, Dashnow H, De Sena Brandine G, Mokveld T, et al. Characterization and visualization of tandem repeats at genome scale. Nat Biotechnol 2024;42:1606–1614.
28. Sivakumar AK, Sudarsanam S, Sharma A, Avvaru AK, Sowpati DT. ATaRVa: Analysis of Tandem Repeat Variation from Long Read Sequencing data. bioRxiv 2025;p. 2025.05.13.653434.
29. Mukamel RE, Handsaker RE, Sherman MA, Barton AR, Zheng Y, et al. Protein-coding repeat polymorphisms strongly shape diverse human phenotypes. Science 2021;373:1499–1505.
30. Bakhtiari M, Park J, Ding YC, Shleizer-Burko S, Neuhausen SL, et al. Variable number tandem repeats mediate the expression of proximal genes. Nat Commun 2021;12:2075.
31. Lu TY, Smaruj PN, Fudenberg G, Mancuso N, Chaisson MJP. The motif composition of variable number tandem repeats impacts gene expression. Genome Res 2023;33:511–524.
32. Cheng H, Concepcion GT, Feng X, Zhang H, Li H. Haplotype-resolved de novo assembly using phased assembly graphs with hifiasm. Nat Methods 2021;18:170–175.
33. Rautiainen M, Nurk S, Walenz BP, Logsdon GA, Porubsky D, et al. Telomere-to-telomere assembly of diploid chromosomes with Verkko. Nat Biotechnol 2023;41:1474–1482.
34. Li H, Bloom JM, Farjoun Y, Fleharty M, Gauthier L, et al. A synthetic-diploid benchmark for accurate variant-calling evaluation. Nat Methods 2018;15:595–597.
35. Hickey G, Monlong J, Ebler J, Novak AM, Eizenga JM, et al. Pangenome graph construction from genome alignments with Minigraph-Cactus. Nat Biotechnol 2024;42:663–673.
36. Garrison E, Guarracino A, Heumos S, Villani F, Bao Z, et al. Building pangenome graphs. Nat Methods 2024;21:2008–2012.

Manuscript with marked changes

Click here to access/download;Manuscript;lcr-sv-marked.pdf

Placeholder for OUP logo oup.pdf

Placeholder for journal logo gigascience-logo.pdf

GigaScience, 2025, 1–5

doi: xx.xxxx/xxxx
Manuscript in Preparation
Data Note

DATA NOTE

Challenges in structural variant calling in low-complexity regions

Qian Qin[1] and Heng Li[2,3,4,*]

[1]Brigham Women's Hospital, 75 Francis St, Boston, MA 02115, USA and [2]Department of Biomedical Informatics, Harvard Medical School, 10 Shattuck St, Boston, MA 02215, USA and [3]Department of Data Science, Dana-Farber Cancer Institute, 450 Brookline Ave, Boston, MA 02215, USA and [4]Broad Insitute of MIT and Harvard, 415 Main St, Cambridge, MA 02142, USA

[*]hli@ds.dfci.harvard.edu

Abstract

Background: Structural variants (SVs) are genomic differences ≥50 bp in length. They remain challenging to detect even with long sequence reads, and the sources of these difficulties are not well quantified.

Results: We identified 35.4 Mb of low-complexity regions (LCRs) in GRCh38. Although these regions cover only 1.2% of the genome, they contain 69.1% of confident SVs in sample HG002. Across long-read SV callers, 77.3–91.3% of erroneous SV calls occur within LCRs, with error rates increasing with LCR length.

Conclusion: SVs are enriched and difficult to call in LCRs. Special care needs to be taken for calling and analyzing these variants.

Key words: structural variant; low-complexity regions; evaluation

Introduction

Structural variants (SVs) are ≥50bp genomic variants and may have functional impacts [1]. Recent work based on high-quality long-read assemblies suggests there are broadly 25,000–35,000 SVs per human individual [2, 3]. Constructed by the Genome-In-A-Bottle (GIAB) group, the latest SV benchmark HG002-Q100 v1.1 [4] contains 28,188 SVs in 2.76Gb of confident regions, consistent with the recent counts. In contrast, published in 2020 [5], the older HG002-SV benchmark v0.6 only contains 9,705 SVs in 2.66Gb. This seems to suggest ~18,000 SVs would fall in ~100Mb regions if we assume the SV v0.6 regions are contained in Q100 v1.1. Is this the correct interpretation?

This article shows that the differences between the two versions of the GIAB SV benchmarks are primarily driven by low-complexity regions (LCRs) that harbor repeatedly occurring motifs. The older benchmark excluded many of LCRs because it was hard to call them correctly. Although SV callers developers have noticed the difficulties in calling SVs around LCRs [5, 6, 7], they have not systematically quantified the effect of LCRs in SV calling. There is not a consensus on the number of SVs in LCRs or the error rate of them. Here, we identified LCRs jointly from the reference genome and the assemblies from the Human Pangenome Reference Consortium (HPRC) [2], and evaluated their impact on SV calling with multiple callers.

Data Description

We applied longdust [8] to GRCh38 and identified 115.4Mb of LCRs on assembled chromosomes. We filtered about half of them that overlap with alpha and HSAT2/3 centromeric repeats found by dna-brnn [9]. 34.4Mb of LCRs were left when we selected LCRs of 50bp or longer.

GRCh38 only represents one human genome. It may miss polymorphic LCRs present in other human samples but missing from GRCh38. To look for these LCRs, we ran longdust on all 462 assemblies from HPRC and used the results to annotate variant bubbles in the minigraph graph of these assemblies [10]. A variant bubble was marked as an LCR if (a) ≥70% of the sequences in the bubble were LCRs in the source assemblies, and (b) the sequences in the bubble were not annotated as segmental duplications (SegDup) by HPRC. Note that if an LCR falls in a long polymorphic SegDup, most of the sequences in the corresponding bubble will be annotated

Compiled on: November 19, 2025.
Draft manuscript prepared by the author.

as SegDup but not as LCR. This is why we put SegDup at a higher priority over LCRs during annotation. To focus on common variants, we dropped non-GRCh38 alleles supported by <5 assemblies. We ignored HG002 when counting alleles because we will use this sample for benchmarking later.

We merged the common polymorphic LCRs and GRCh38 LCRs and added 5bp to both ends of each LCR because LCR boundaries may not be exact and by convention, insertions are often placed right before exact tandem repeats. This resulted in a BED file with 111,067 records, covering 35.4Mb of GRCh38. 29,291 records overlap with common polymorphic LCRs in the HPRC minigraph graph. 3,918 of them are not observed on GRCh38. 16.2% of the LCRs are intersected with the SegDup annotation from the "genomicSuperDups" track of the UCSC Genome Browser [11]. We see the overlap because an LCR consisting of several copies of a long repeat unit could also be considered as a SegDup.

LCRs are closely related to tandem repeats. Longdust identifies most tandem repeats with ≥4 copies of repeat units as LCRs [10], but it often misses tandem repeats with fewer copies and may report additional regions without clear tandem patterns. 83.5% of our LCRs overlap with tandem repeats found by TRF [12] v4.10 (option `2 7 7 80 10 50 500 -l12`) and 92.0% overlap with TR Catalog [13] v1.2.1 which covers 238Mb of GRCh38, much larger than our regions.

We applied the same procedure to the T2T-CHM13 genome [14] and found 79.6Mb of LCRs, doubling the length of LCRs in GRCh38. Most of the additional regions came from centromeric satellites that are not HSAT2/3 or alpha repeats. If we exclude all types of satellites [15], only 31.2Mb is left. The remaining difference in size from GRCh38 LCRs is probably caused by satellite annotation.

Data Analysis

To understand the effect of LCRs in long-read SV calling, we measured the accuracy of SV calls stratified by LCR. We called SVs with 11 callers and compared them to both the new HG002-Q100 v1.1 [4] and the old HG002-SV v0.6 [5] benchmarks to demonstrate the impact of LCR in SV calling.

Investigating the GIAB truth SVs

There are 29,131 SVs of ≥50bp in length contained in the confident regions in the new HG002-Q100 v1.1 benchmark [4]. 943 of them have "*" as alternate alleles. We manually inspected the read alignment around some of these SVs and believe they are all redundant. Removing them from the truth left us with 28,188 SVs. The truvari [16] evaluation tool also filters SVs with "*" alleles.

The older HG002-SV v0.6 benchmark [5] is only available in the GRCh37 coordinate. To evaluate the SV calling accuracy on this benchmark, we lifted its confident regions over to GRCh38 with UCSC's liftover web service which failed on 0.03% of intervals. We still took SVs from HG002-Q100 as the ground truth. There are 11,985 HG002-Q100 overlapping with the lifted HG002-SV confident regions, more than the 9,705 SVs from the older HG002-SV benchmark. The difference is caused by the allele resolution. Suppose both haplotypes in HG002 harbor a 6kb insertion to the same location of the reference genome. The inserted sequences however differ by one SNP between them. The newer HG002-Q100 benchmark would consider this event as two heterozygous insertions, but the older HG002-SV benchmark would merge the two insertion alleles and consider them as one homozygous insertion. As a result, we counted 7,362 insertions in HG002-Q100 v1.1 but only 5,444 in HG002-SV v0.6, a sharp reduction. At the same time, the allele resolution may also affect deletions. If there are overlapping deletions of similar lengths between the two haplotypes, HG002-Q100 will encode them two independent deletions, but HG002-SV may merge them and thus reduce the total counts. Overall, constructed from long-read assemblies, HG002-Q100 is more precise and more accurate than HG002-SV.

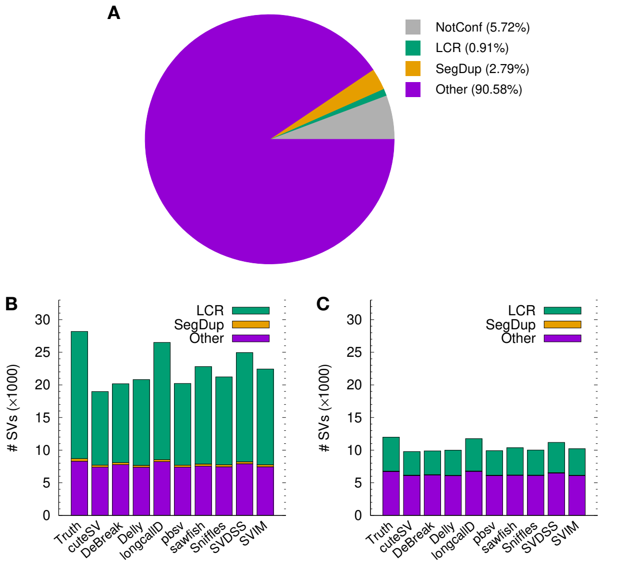

Figure 1. Number of HG002 structural variants (SVs) on GRCh38. (A) Lengths of regions. "NotConf" denotes not-confident regions in the HG002-Q100 v1.1 benchmark, excluding assembly gaps in GRCh38. A region classified to a previous type will not be counted towards the next type in the order of NotConf, LCR (low-complexity region), SegDup (segmental duplication) and Other. (B) Number of HG002-Q100 SVs stratified by LCR, SegDup and the rest of the confident regions. An SV is classified as LCR (or SegDup) if ≥70% of its interval on GRCh38 overlaps with LCR (or SegDup). An SV classified as LCR will not be classified as SegDup. (C) Number of HG002-Q100 SVs in the HG002-SV v0.6 confident regions lifted over from GRCh37.

Calling SVs from long reads

We acquired PacBio High-Fidelity reads from HPRC [17], aligned them to the primary assembly of GRCh38 with minimap2 [18] and called SVs with cuteSV v2.1.1 [19], DeBreak v1.0.2 [20], Delly v1.3.3 [21], longcallD v0.0.5 [22], pbsv v2.11.0 [23], Sawfish v0.12.10 [24], Sniffles2 v2.6.3 [6], SVDSS v2.1.0 [25], and SVIM v2.0.0 [26]. We used kanpig v1.1.0 [13] for genotyping SVs called by SVDSS as is suggested in the documentation. Sniffles2 may optionally take tandem repetitive regions as input, but using this option slightly reduced its overall accuracy, so we only evaluated its default setting.

We also tried specialized tandem repeat callers including TRGT [27] and ATaRVa [28]. These tools may output both long reference and long alternate alleles for one variant. Truvari was unable to correctly evaluate such variants and greatly overestimated false positives. We thus did not include tandem repeat callers in this work.

Most SVs are located in LCRs

We stratified HG002 SVs by LCR and SegDup (Fig. 1). For an SV to be classified as LCR or SegDup, we required it to have large overlap with LCR or SegDup regions. Without this condition, a long deletion containing a short LCR would be falsely classified as LCR, which would inflate the number of LCR SVs. Across the SV callers, 59.4–67.7% of SV calls overlap with LCR, although LCR only accounts for 1.2% of GRCh38 or 0.9% of GIAB confident regions. SVs are highly enriched in LCR. On the ground truth, SVs in LCR contributed to 42.1% of total SV lengths. This suggests these SVs are shorter than the average.

Whereas the numbers of "Other" SVs in the HG002-Q100 confident regions are similar across callers, the numbers of LCR SVs differ greatly (Fig. 1B). SV callers that attempt to produce haplotype-

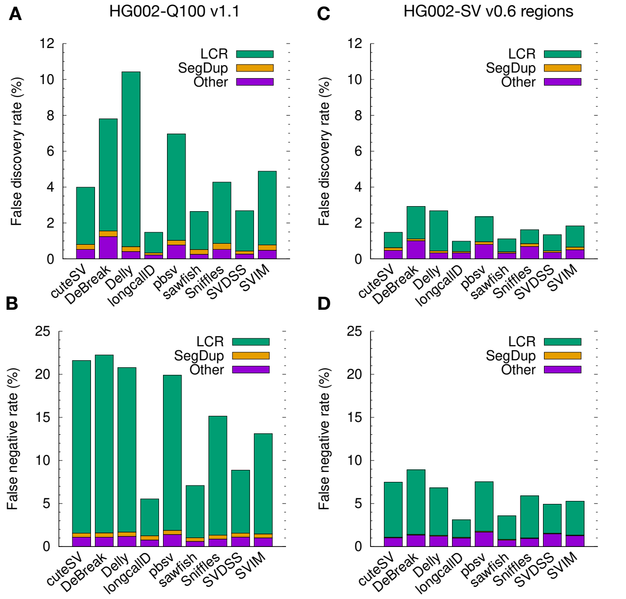

Figure 2. Accuracy of SV calls. (A) False discovery rate (FDR) of SVs in the HG002-Q100 confident regions, measured by truvari in the "refine" mode. SVs are stratified to LCR, SegDup and Other in the same way as is described in Fig. 1. (B) False negative rate (FNR) of SVs in HG002-Q100. (C) FDR in the HG002-SV confident regions. (D) FNR in HG002-SV.

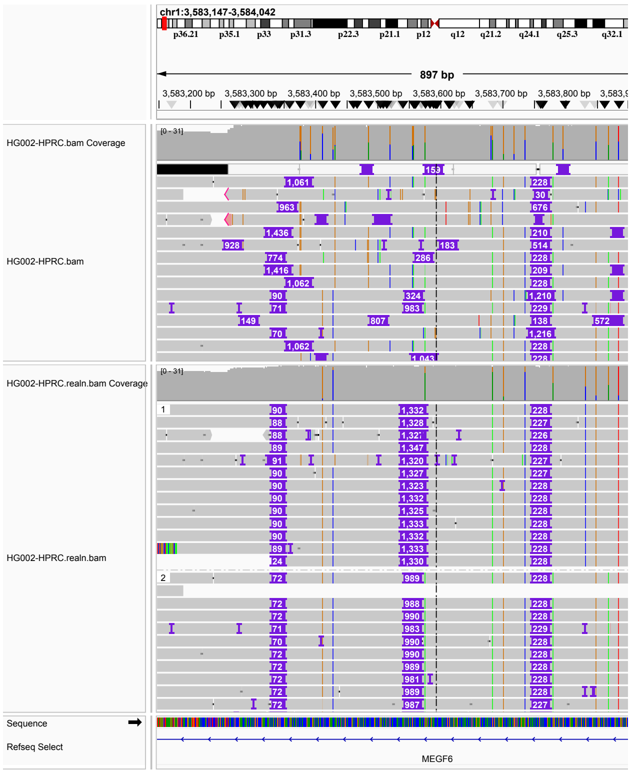

Figure 3. IGV screenshot of alignment around an LCR. The top panel shows the raw alignment by minimap2. The bottom panel shows the phased realignment by longcallD. There are 1650 (=90+1332+228) inserted bases on the first haplotype in total and 1290 (=72+990+228) inserted bases on the second haplotype, identical to the HG002-Q100 ground truth.

resolved SVs, such as longcallD and SVDSS, call noticeably more SVs in LCR and SegDup. This trend is also observed in the older HG002-SV v0.6 confident regions (Fig. 1C). In the older HG002-SV regions, there are much fewer SVs in LCR and almost none in SegDup, although the numbers of SVs in Other regions are only reduced a little. This indicates that the main difference between HG002-Q100 and HG002-SV comes from LCRs.

SVs in LCRs are harder to call correctly

We evaluated SV calls with truvari v5.3.0 [16], which performs multi-sequence alignment to normalize different variant representations and is recommended by GIAB. Having explored multiple truvari options, we settled on "`bench --passonly --pick ac --dup-to-ins`" followed by "`refine --use-original-vcfs`" as the resulting accuracy matched our manual inspection better.

On the new HG002-Q100 benchmark, 31.1–39.0% of SVs, depending on callers, are marked as "Other" (Fig. 1B), but only 5.2–14.0% of SV errors come from "Other" (Fig. 2A and 2B). This suggests SVs in the Other category are easier to call. In contrast, the majority of errors, at 77.3–91.3%, are located in LCRs. SVs in SegDup are also difficult to call, but due to the small number of such SVs, they do not contribute much to the total number of errors.

Developed in our group but unpublished, longcallD achieves the lowest error rate (Fig. 2) mainly because it performs haplotype-aware multi-sequence realignment. As is shown in the top panel of Fig. 3, minimap2 often places gaps differently across reads and thus produces inconsistent alignment in long LCRs. This happens because minimap2 does not see other reads in the same region during pairwise alignment. It is not apparent that there are only two haplotypes in this region. Such inconsistency would confuse most SV callers. For this example, the SV callers in the order shown in Fig. 2, respectively, called +1007/+1007, +1392/+1392, +1293/+1293, +1650/+1290, +1278/+1278, +1278/+1668, +963/+1191, +1650/+1290 and +1353/+2306 insertions on the two haplotypes. Only longcallD and SVDSS found the precise allele lengths of +1650/+1290. Nonetheless, truvari considered all callers correct. The error rate of most callers would probably be higher if we required precise allele matches.

We further stratified the errors by the maximum allele length of each LCR (Fig. 4) and observed increased error rates with maximum allele lengths. Some callers missed about half of SVs in ≥2kb LCRs, even though HiFi reads are long enough to span most them. Simple algorithms without realignment or reassembly are not capable of calling SVs in long LCRs.

Discussion

LCR SVs are a distinct class. Although LCRs only contribute to 1.2% of GRCh38 excluding alpha and HSAT2/3 repeats, they harbor more than half of long-read SV calls and an even higher fraction of SV calling errors. These errors are mainly caused by inconsistent read alignment especially around long LCRs. Short-read SV calling may be affected more due to uncertainty in alignment around LCRs.

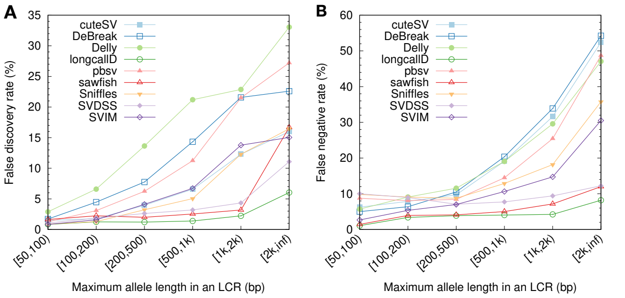

Figure 4. Accuracy of SV calls stratified by the maximum allele length in LCR. If an LCR is a common polymorphism (supported by ≥5 non-GRCh38 assemblies in HPRC), the maximum allele length equals to the length of the longest allele aligned to the LCR; otherwise, the maximum allele length equals to the length of the LCR on GRCh38.

On the other hand, we note that LCRs may overlap with coding exons of genes that have functional impacts [29] and they may also mediate gene expression [30, 31]. We would not want to filter all SVs overlapping LCRs.

For data analysts, we recommend stratifying SVs by LCR as LCR SVs are enriched with errors and are resulted from different biological processes. For developers, we would like to emphasize the critical role of realignment or local reassembly in accurate SV calling. Most SVs in LCRs can still be called to decent accuracy with good algorithms.

Given accurate long reads at high coverage, we may also assemble the reads with haplotype-resolved assemblers [32, 33] and call variants from assembly-to-reference alignment [34]. Performing phasing and alignment within each haplotype, these assemblers are more powerful than most SV callers. As a matter of fact, the HG002-Q100 truth was derived this way.

We have only analyzed one human sample in this article. If mainstream SV callers are already struggling with long LCRs, merging their calls across different samples will be more problematic. When haplotype-resolved assembly is possible, calling variants across samples with pangenome-based methods [10, 35, 36] will be the preferred approach as conducting multi-sequence alignment across samples, such methods can produce more consistent SV representations. They may also struggle with highly variable LCRs, but will do better than traditional SV merging in most cases.

Data Availability

LCRs are available at `https://doi.org/10.5281/zenodo.10903864` (file "`chm13v2.lcr-v4.bed.gz`" and "`hg38.lcr-v4.bed.gz`"). Scripts used for producing the LCRs and plots can be found at `https://github.com/lh3/lcr-sv`.

Declarations

List of abbreviations

GIAB: Genome-In-A-Bottle group; HPRC: Human Pangenome Reference Consortium; kb: kilobase; LCR: low-complexity regions; Mb: megabase; SegDup: segmental duplication; SV: structural variant.

Competing Interests

The authors declare they have no competing interests.

Funding

This work is supported by National Institute of Health grant R01HG010040, R01HG014175, U24CA294203, U01HG013748 and U41HG010972 (to H.L.).

Author's Contributions

H.L. conceived the project. Q.Q. produced structural variant calls. Q.Q. and H.L. analyzed the data and drafted the manuscript.

Acknowledgements

We would like to acknowledge the National Genome Research Institute (NHGRI) for funding the following grants supporting the creation of the human pangenome reference: U41HG010972, U01HG010971, U01HG013760, U01HG013755, U01HG013748, U01HG013744, R01HG011274, and the Human Pangenome Reference Consortium (BioProject ID: PRJNA730823).

References

1. Eichler EE. Genetic Variation, Comparative Genomics, and the Diagnosis of Disease. N Engl J Med 2019;381:64–74.
2. Liao WW, Asri M, Ebler J, Doerr D, Haukness M, et al. A draft human pangenome reference. Nature 2023;617:312–324.
3. Logsdon GA, Ebert P, Audano PA, Loftus M, Porubsky D, et al. Complex genetic variation in nearly complete human genomes. Nature 2025;644:430–441.
4. Hansen NF, Dwarshuis N, Ji HJ, Rhie A, Loucks H, et al. A complete diploid human genome benchmark for personalized genomics. bioRxiv 2025;p. 2025.09.21.677443.
5. Zook JM, Hansen NF, Olson ND, Chapman L, Mullikin JC, et al. A robust benchmark for detection of germline large deletions and insertions. Nat Biotechnol 2020;38:1347–1355.
6. Smolka M, Paulin LF, Grochowski CM, Horner DW, Mahmoud M, et al. Detection of mosaic and population-level structural variants with Sniffles2. Nat Biotechnol 2024;42:1571–1580.
7. Keskus AG, Bryant A, Ahmad T, Yoo B, Aganezov S, et al. Severus detects somatic structural variation and complex rearrangements in cancer genomes using long-read sequencing. Nat Biotechnol 2025;.
8. Li H, Li B. Finding low-complexity DNA sequences with longdust. ArXiv 2025;p. arXiv:2509.07357v1.
9. Li H. Identifying centromeric satellites with dna-brnn. Bioinformatics 2019;35:4408–4410.
10. Li H, Feng X, Chu C. The design and construction of reference pangenome graphs with minigraph. Genome Biol 2020;21:265.
11. Perez G, Barber GP, Benet-Pages A, Casper J, Clawson H, et al. The UCSC Genome Browser database: 2025 update. Nucleic Acids Res 2025;53:D1243–D1249.
12. Benson G. Tandem repeats finder: a program to analyze DNA sequences. Nucleic Acids Res 1999;27:573–80.
13. English AC, Cunial F, Metcalf GA, Gibbs RA, Sedlazeck FJ. K-mer analysis of long-read alignment pileups for structural variant genotyping. Nat Commun 2025;16:3218.
14. Nurk S, Koren S, Rhie A, Rautiainen M, Bzikadze AV, et al. The complete sequence of a human genome. Science 2022;376:44–53.
15. Altemose N, Logsdon GA, Bzikadze AV, Sidhwani P, Langley SA, et al. Complete genomic and epigenetic maps of human centromeres. Science 2022;376:eabl4178.
16. English AC, Menon VK, Gibbs RA, Metcalf GA, Sedlazeck FJ. Truvari: refined structural variant comparison preserves allelic diversity. Genome Biol 2022;23:271.
17. Human Pangenome Reference Consortium;. `https://human-pangenomics.s3.amazonaws.com/submissions/80d00e88-7a92-46d8-88c7-48f1486e11ed--HG002_PACBIO_REVIO/m84039_230117_233243_s1.hifi_reads.default.bam`.
18. Li H. Minimap2: pairwise alignment for nucleotide sequences. Bioinformatics 2018;34:3094–3100.
19. Jiang T, Liu Y, Jiang Y, Li J, Gao Y, et al. Long-read-based human genomic structural variation detection with cuteSV. Genome Biol 2020;21:189.
20. Chen Y, Wang AY, Barkley CA, Zhang Y, Zhao X, et al. Deciphering the exact breakpoints of structural variations using long sequencing reads with DeBreak. Nat Commun 2023;14:283.
21. Rausch T, Zichner T, Schlattl A, Stütz AM, Benes V, Korbel JO. DELLY: structural variant discovery by integrated paired-end and split-read analysis. Bioinformatics 2012;28:i333–i339.
22. ;. `https://github.com/yangao07/longcallD`.
23. ;. `https://github.com/PacificBiosciences/pbsv`.
24. Saunders CT, Holt JM, Baker DN, Lake JA, Belyeu JR, et al. Sawfish: improving long-read structural variant discovery and genotyping with local haplotype modeling. Bioinformatics 2025;41:btaf136.
25. Denti L, Khorsand P, Bonizzoni P, Hormozdiari F, Chikhi R. SVDSS: structural variation discovery in hard-to-call genomic regions using sample-specific strings from accurate long reads.

Nat Methods 2023;20:550–558.
26. Heller D, Vingron M. SVIM: structural variant identification using mapped long reads. Bioinformatics 2019;35:2907–2915.
27. Dolzhenko E, English A, Dashnow H, De Sena Brandine G, Mokveld T, et al. Characterization and visualization of tandem repeats at genome scale. Nat Biotechnol 2024;42:1606–1614.
28. Sivakumar AK, Sudarsanam S, Sharma A, Avvaru AK, Sowpati DT. ATaRVa: Analysis of Tandem Repeat Variation from Long Read Sequencing data. bioRxiv 2025;p. 2025.05.13.653434.
29. Mukamel RE, Handsaker RE, Sherman MA, Barton AR, Zheng Y, et al. Protein-coding repeat polymorphisms strongly shape diverse human phenotypes. Science 2021;373:1499–1505.
30. Bakhtiari M, Park J, Ding YC, Shleizer-Burko S, Neuhausen SL, et al. Variable number tandem repeats mediate the expression of proximal genes. Nat Commun 2021;12:2075.
31. Lu TY, Smaruj PN, Fudenberg G, Mancuso N, Chaisson MJP. The motif composition of variable number tandem repeats impacts gene expression. Genome Res 2023;33:511–524.
32. Cheng H, Concepcion GT, Feng X, Zhang H, Li H. Haplotype-resolved de novo assembly using phased assembly graphs with hifiasm. Nat Methods 2021;18:170–175.
33. Rautiainen M, Nurk S, Walenz BP, Logsdon GA, Porubsky D, et al. Telomere-to-telomere assembly of diploid chromosomes with Verkko. Nat Biotechnol 2023;41:1474–1482.
34. Li H, Bloom JM, Farjoun Y, Fleharty M, Gauthier L, et al. A synthetic-diploid benchmark for accurate variant-calling evaluation. Nat Methods 2018;15:595–597.
35. Hickey G, Monlong J, Ebler J, Novak AM, Eizenga JM, et al. Pangenome graph construction from genome alignments with Minigraph-Cactus. Nat Biotechnol 2024;42:663–673.
36. Garrison E, Guarracino A, Heumos S, Villani F, Bao Z, et al. Building pangenome graphs. Nat Methods 2024;21:2008–2012.

A1.0.1: Reviewers asked us about the relationship between LCRs and tandem repeats (TRs). We discussed in detail in our longdust preprint. We have added a short summary in this revision: "*LCRs are closely related to tandem repeats. Longdust identifies most tandem repeats with ≥4 copies of repeat units as LCRs [10], but it often misses tandem repeats with fewer copies and may report additional regions without clear tandem patterns. 83.5% of our LCRs overlap with tandem repeats found by TRF [12] v4.10 (option 2 7 7 80 10 50 500 -l12) and 92.0% overlap with TR Catalog [13] v1.2.1 which covers 238Mb of GRCh38, much larger than our regions.*" We use longdust instead of TRF because longdust is >10 times faster and we understand the behavior of longdust better.

A1.0.2: Reviewers also suggested running tandem repeat callers like TRGT. We had run these tools in early stage but observed low accuracy. We now explain our finding in the revised manuscript: "*We also tried specialized tandem repeat callers including TRGT [27] and ATaRVa [28]. They may output both long reference and long alternate alleles for one variant. Truvari was unable to correctly evaluate such variants and greatly overestimated false positives. We thus did not include tandem repeat callers in this work.*"

In general, SV calling papers and tandem repeat calling papers use distinct methods to evaluate accuracy. Although TRsv (Kosugi and Terao, 2025) evaluates both types of variants, its SV evaluation method is relatively simple. Dedicated tools like truvari are much more sophisticated at unifying different SV representations.

Reviewer #1

Summary Statement
This manuscript addresses an important and timely topic in genome analysis, and the authors' effort to systematically examine low-complexity regions is commendable. The study has the potential to contribute valuable insights into the challenges of variant interpretation in difficult genomic contexts. However, several critical analyses are missing, and the presentation would benefit from significant reorganization and clarification. In particular, the relationship between tandem repeats and low-complexity regions is not sufficiently explored, which limits the interpretability and completeness of the findings. With additional analyses and improved focus, this work could make a meaningful contribution to the field.

Major Comments

Q1.1.1: 1. Incomplete treatment of tandem repeats (TRs)
The manuscript does not sufficiently address the overlap between low-complexity sequences and tandem repeats (TRs). TRs often coincide with low-complexity regions, small indel variants, and structural variation, making them an important genomic feature to consider. Without incorporating TR context, the analyses risk being oversimplified. For example, in Figure 3, the depicted region corresponds to an entry in the Adotto tandem repeat catalog (chr1:3583240-3583962; https://github.com/ACEnglish/adotto). The authors should clarify how such regions were handled and whether TRs were explicitly excluded, annotated, or analyzed separately.

A1.1.1: Please see **A1.0.1** on LCR vs TR. Our project in fact started with the Adotto TR Catalog. However, we noticed many regions do not look low complexity visually. For example, the FNR of Sniffles SVs overlapping with our regions is 20%; the Sniffles FNR outside our regions but inside TR Catalog is 3.6%, while the FNR of the rest of non-segdup SVs is 3.0%. This suggests TR Catalog outside our regions is not noticeably more difficult for SV calling. TR Catalog is developed for general STR calling but not for our purpose. The analysis above would be unfair to TR Catalog, so we did not show it in our manuscript.

Q1.1.2: 2. Performance of TR/VNTR callers in low-complexity regions
A natural extension of the above point concerns how tandem repeat or VNTR-specific tools (for example, TRGT [Dolzhenko], Vamos [Chaisson], and others) would perform within these "LCRs." Many of these regions are, in fact, tandem repeats, and it would strengthen the manuscript to discuss or benchmark the performance of these specialized tools in that context. Even a qualitative discussion would add depth and help position the current work within the broader methodological landscape.

A1.1.2: Please see **A1.0.2** on why we have not reported TRGT results.

Q1.1.3: 3. Quantification of variation by sequence length
The manuscript primarily reports counts of variants, particularly structural variants, without contextualizing them by the amount of sequence affected. Given that a typical human genome differs from the reference by roughly 20 Mb of structural variation, it would be more informative to describe results in terms of sequence length impacted rather than event counts alone. This approach would better reflect the biological impact and improve interpretability of the results.

A1.1.3: We added that "*On the ground truth, SVs in LCR contributed to 42.1% of total SV lengths. This suggests these SVs are shorter than the average*". We note that most SV calling papers count the number of events because multi-base changes, at least mobile element insertions, were often caused by one event in evolution.

Q1.1.4: 4. Clarity and structure of the prose
The writing would benefit from careful editing to improve clarity and logical flow. At present, it can be difficult to identify the central findings and their supporting evidence. The authors are encouraged to restructure the manuscript to guide the reader more clearly through the rationale, methods, results, and conclusions.
Specific examples include:
o "This article gives the answer" is conversational and not typical for scientific literature.
o "We will also explain caveats in the truth data" has an unclear meaning and seems incomplete.
o "Developed in our group" should be revised, as LongCallD does not appear to be a published or preprinted method. To avoid confusion, the authors might clarify the status of the tool or use more neutral phrasing.

A1.1.4: We changed "This article gives the answer" to "*This article shows that*". We removed "We will also explain caveats in the truth data". We changed "Developed in our group" to

“*Developed in our group but unpublished*”. We mention our involvement to let readers beware of potential biases towards our own tool.

Reviewer #2

The authors delineate low-complexity regions (LCRs) and show that although LCRs comprise only ~1.2% of GRCh38, they harbor the majority of confident SVs and the vast majority of calling errors across long-read callers; error rates rise with LCR length.

Major comments

Q1.2.1: 1. You report 79.6 Mb of LCRs on T2T-CHM13 that drop to 31.2 Mb after excluding satellites, versus 35.4 Mb on GRCh38. If T2T-CHM13 truly "resolves" specific GRCh38 LCRs, please add 1-2 concrete locus examples to illustrate what is gained.

A1.2.1: T2T-CHM13 should have similar amount of LCRs outside centromeres. We now explain that “*The remaining difference in size from GRCh38 LCRs is probably caused by satellite annotation.*”

Q1.2.2: 2. Please detail the GRCh37→GRCh38 liftover for the v0.6 regions. Did you lift over entire intervals or only start and end coordinates? Also, how many SVs (and what proportion) failed liftover?

A1.2.2: We now say “*we lifted its confident regions over to GRCh38 with UCSC’s liftover web service which failed on 0.03% of intervals*”. UCSC liftover lifts regions, not just start and end.

Q1.2.3: 3. Do you expect the conclusions to hold if reads are aligned to T2T-CHM13 instead of GRCh38?

A1.2.3: The conclusion will stay the same. Most LCRs outside centromeric satellites are not difficult to assemble. T2T-CHM13 would not make a big difference.

Q1.2.4: 4. Because most public biobank data are short-read, LCRs are likely to exacerbate ambiguity, so both FNR and FDR may be higher than with long reads. Please add a brief note in the Discussion to set expectations and, if feasible, a small comparative run on HG002 using a standard short-read SV pipeline to show whether the LCR error gradient is amplified.

A1.2.4: We added in Discussion that “*Short-read SV calling may be affected more due to uncertainty in alignment around LCRs*”.

Minor comment

Q1.2.5: 5. Abstract/Conclusion: "Special care need to be taken …" → "Special care needs to be taken …".

A1.2.5: Fixed. We thank the reviewer for the correction.

Reviewer #3

This study reports a high error calling rate for structural variations (SVs) detected with long read data in low complexity regions (LCRs). The representation of the results appears valid, but the information regarding the accuracy of SV calling is insufficient.

Major points:

Q1.3.1: 1. This study focused on analyzing SV calling accuracy in LCRs, defined as regions that harbor repeatedly occurring motifs. The authors should provide a more detailed explanation of LCR: clarifying whether LCRs include tandem repeats (TRs) such as short tandem repeats (STRs) and variable number of tandem repeats (VNTRs). If there are differences from general TR, they should describe those differences. If LCRs defined in this study do not include inverted repeats, the term TR may be more appropriate than LCR.
The LCRs used in this study were identified using the logdust tool developed by the authors. It is necessary to explain the differences between TRs identified using TRF (e.g., a simpleRepeat.txt file available on the UCSC site) and LCRs.

A1.3.1: Please see **A1.0.1** on TRs vs LCRs.

Q1.3.2: 2. This study presents false discovery rate (FDR) and false negative rate (FNR) for SV calling (Figs. 2 and 4). However, it does not provide information on the type of errors causing high FDR and FNR in LCRs (e.g., errors in SV length or breakpoint distance between SV calls and reference SVs). Given that this study focuses on SV detection accuracy in LCRs, this information is essential.

A1.3.2: SV comparison is non-trivial. We rely on truvari for evaluation and refer readers to its paper (English et al, 2022) for technical details. Truvari is considered as the state of the art for SV evaluation. In the main text, we now mention: "*[truvari] performs multi-sequence alignment to normalize different variant representations and is recommended by GIAB*".

Q1.3.3: 3. Within the TR region, which constitutes primary components of LCRs, insertions and deletions composed of the TR repeat units can be assigned to any position within the TR region. For example, in a 200 bp TR region consisting of 20 copies of a 10 bp repeat unit, a 100 bp insertion consisting of 10 copies of the same repeat unit could be potentially aligned at the beginning, middle, or end of the TR region in a long read or assembly alignment. In addition, as shown in Fig. 3 and a previous report (Genome Biol 26, 246 2025), an insertion sequence composed of identical or similar repeat units could be fragmented into multiple insertion sequences within a TR region in a long read or assembly alignment. Therefore, SVs may be assigned to different positions within an LCR depending on alignment tools, alignment parameters, and read/assembly data. That is true for the HG002-Q100 truth SV data, which is derived from the alignments of haplotype-resolved assemblies to GRCh38. The results in Fig. 4 likely reflect this effect. The use of TR genotyping tools, such as LongTR and

TRGT, may resolve this issue. These points should be discussed in the manuscript.

A1.3.3: We now mention “*minimap2 often places gaps differently across reads*”. We want to emphasize that in LCRs, the main problem with most existing SV callers is not where to place INDELs (with MSA, truvari can greatly alleviate the issue); it is that they report wrong total allele lengths for examples like Fig. 3. We agree with the reviewer that tandem repeat callers may have an advantage here. We explained why we did not show their results in **A1.0.2**.

Minor point:

Q1.3.4: In line 8 on page 2, 'added 5bp to both ends of each LCR': What is the rationale for adding 5 bp to both ends of each LCR?

A1.3.4: We have changed the sentence to “*[we] added 5bp to both ends of each LCR because LCR boundaries may not be exact and by convention, insertions are often placed right before exact tandem repeats*”.
