# Supplementary material for: Challenges in structural variant calling in low-complexity regions
Source: Gigascience. 2025 Dec 12;14:giaf154. doi: 10.1093/gigascience/giaf154 (PMC12758381; doi:10.1093/gigascience/giaf154)
Supplement: giaf154_GIGA-D-25-00409_Revision_2 [file giaf154_giga-d-25-00409_revision_2.pdf]

GigaScience

Challenges in structural variant calling in low-complexity regions

--Manuscript Draft--

**Manuscript Number:**	GIGA-D-25-00409R2
**Full Title:**	Challenges in structural variant calling in low-complexity regions
**Article Type:**	Data Note
**Funding Information:**	National Human Genome Research Institute (R01HG010040) — Dr. Heng Li<br>National Human Genome Research Institute (R01HG014175) — Dr. Heng Li<br>National Human Genome Research Institute (U01HG013748) — Dr. Heng Li<br>National Human Genome Research Institute (U41HG010972) — Dr. Heng Li<br>National Cancer Institute (US) (U24CA294203) — Dr. Heng Li
**Abstract:**	Background: Structural variants (SVs) are genomic differences ≥50 bp in length. They remain challenging to detect even with long sequence reads, and the sources of these difficulties are not well quantified.<br>Results: We identified 35.4 Mb of low-complexity regions (LCRs) in GRCh38. Although these regions cover only 1.2% of the genome, they contain 69.1% of confident SVs in sample HG002. Across long-read SV callers, 77.3–91.3% of erroneous SV calls occur within LCRs, with error rates increasing with LCR length.<br>Conclusion: SVs are enriched and difficult to call in LCRs. Special care needs to be taken for calling and analyzing these variants.
**Corresponding Author:**	Heng Li<br>Dana-Farber Cancer Institute<br>Boston, UNITED STATES
**Corresponding Author Secondary Information:**	
**Corresponding Author's Institution:**	Dana-Farber Cancer Institute
**Corresponding Author's Secondary Institution:**	
**First Author:**	Qian Qin
**First Author Secondary Information:**	
**Order of Authors:**	Qian Qin
	Heng Li
**Order of Authors Secondary Information:**	
**Response to Reviewers:**	Responses to reviewers' comments are uploaded as a PDF file
**Additional Information:**	
**Question**	**Response**
Are you submitting this manuscript to a special series or article collection?	No
**Experimental design and statistics**	Yes

Powered by Editorial Manager® and ProduXion Manager® from Aries Systems Corporation

Full details of the experimental design and statistical methods used should be given in the Methods section, as detailed in our Minimum Standards Reporting Checklist. Information essential to interpreting the data presented should be made available in the figure legends.<br><br>Have you included all the information requested in your manuscript?	
**Resources**<br><br>A description of all resources used, including antibodies, cell lines, animals and software tools, with enough information to allow them to be uniquely identified, should be included in the Methods section. Authors are strongly encouraged to cite Research Resource Identifiers (RRIDs) for antibodies, model organisms and tools, where possible.<br><br>Have you included the information requested as detailed in our Minimum Standards Reporting Checklist?	Yes
**Availability of data and materials**<br><br>All datasets and code on which the conclusions of the paper rely must be either included in your submission or deposited in publicly available repositories (where available and ethically appropriate), referencing such data using a unique identifier in the references and in the "Availability of Data and Materials" section of your manuscript.<br><br>Have you have met the above requirement as detailed in our Minimum Standards Reporting Checklist?	Yes
GigaScience has policies and guidelines in place for the use of generative AI-writing tools such as ChatGPT. If you have used such writing tools to assist with	No

writing the manuscript this must be declared and cited in the text. Authors should not list AI-writing tools and other AI-assisted technologies as an author or co-author and should acknowledge that they are fully responsible for text generated or refined by AI-writing tools.<p><br><br>A summary of use (particularly in the introduction or among methods) needs to be included at the end of the paper, and the outputs should also be included as a supplementary file hosted in GigaDB or other open repositories. Please read our guidelines for more information. <p><br><br>By submitting to GigaScience, you are aware of the journal's AI-writing tools policy, and if you have declared use of such tools below, you have acknowledged this where appropriate in your manuscript and have made a summary of use and outputs available. <p><br><b>AI-assisted writing tools have been used in the preparation of this manuscript?	

LaTeX -Main Document(TeX file)

Click here to access/download;LaTeX - Main Document (TeX file);lcr-sv.tex

Placeholder for OUP logo oup.pdf

Placeholder for journal logo gigascience-logo.pdf

GigaScience, 2025, 1–5

doi: xx.xxxx/xxxx
Manuscript in Preparation
Data Note

DATA NOTE

Challenges in structural variant calling in low-complexity regions

Qian Qin[1] and Heng Li[2,3,4,*]

[1]Brigham Women's Hospital, 75 Francis St, Boston, MA 02115, USA and [2]Department of Biomedical Informatics, Harvard Medical School, 10 Shattuck St, Boston, MA 02215, USA and [3]Department of Data Science, Dana-Farber Cancer Institute, 450 Brookline Ave, Boston, MA 02215, USA and [4]Broad Insitute of MIT and Harvard, 415 Main St, Cambridge, MA 02142, USA

*hli@ds.dfci.harvard.edu

Abstract

Background: Structural variants (SVs) are genomic differences ≥50 bp in length. They remain challenging to detect even with long sequence reads, and the sources of these difficulties are not well quantified.

Results: We identified 35.4 Mb of low-complexity regions (LCRs) in GRCh38. Although these regions cover only 1.2% of the genome, they contain 69.1% of confident SVs in sample HG002. Across long-read SV callers, 77.3–91.3% of erroneous SV calls occur within LCRs, with error rates increasing with LCR length.

Conclusion: SVs are enriched and difficult to call in LCRs. Special care needs to be taken for calling and analyzing these variants.

Key words: structural variant; low-complexity regions; evaluation

Introduction

Structural variants (SVs) are ≥50bp genomic variants and may have functional impacts [1]. Recent work based on high-quality long-read assemblies suggests there are broadly 25,000–35,000 SVs per human individual [2, 3]. Constructed by the Genome-In-A-Bottle (GIAB) group, the latest SV benchmark HG002-Q100 v1.1 [4] contains 28,188 SVs in 2.76Gb of confident regions, consistent with the recent counts. In contrast, published in 2020 [5], the older HG002-SV benchmark v0.6 only contains 9,705 SVs in 2.66Gb. This seems to suggest ~18,000 SVs would fall in ~100Mb regions if we assume the SV v0.6 regions are contained in Q100 v1.1. Is this the correct interpretation?

This article shows that the differences between the two versions of the GIAB SV benchmarks are primarily driven by low-complexity regions (LCRs) that harbor repeatedly occurring motifs. The older benchmark excluded many of LCRs because it was hard to call them correctly. Although SV callers developers have noticed the difficulties in calling SVs around LCRs [5, 6, 7], they have not systematically quantified the effect of LCRs in SV calling. There is not a consensus on the number of SVs in LCRs or the error rate of them. Here, we identified LCRs jointly from the reference genome and the assemblies from the Human Pangenome Reference Consortium (HPRC) [2], and evaluated their impact on SV calling with multiple callers.

Data Description

We applied longdust [8] to GRCh38 and identified 115.4Mb of LCRs on assembled chromosomes. We filtered about half of them that overlap with alpha and HSAT2/3 centromeric repeats found by dna-brnn [9]. 34.4Mb of LCRs were left when we selected LCRs of 50bp or longer.

GRCh38 only represents one human genome. It may miss polymorphic LCRs present in other human samples but missing from GRCh38. To look for these LCRs, we ran longdust on all 462 assemblies from HPRC and used the results to annotate variant bubbles in the minigraph graph of these assemblies [10]. A variant bubble was marked as an LCR if (a) ≥70% of the sequences in the bubble were LCRs in the source assemblies, and (b) the sequences in the bubble were not annotated as segmental duplications (SegDup) by HPRC. Note that if an LCR falls in a long polymorphic SegDup, most of the sequences in the corresponding bubble will be annotated

Compiled on: December 11, 2025.
Draft manuscript prepared by the author.

as SegDup but not as LCR. This is why we put SegDup at a higher priority over LCRs during annotation. To focus on common variants, we dropped non-GRCh38 alleles supported by <5 assemblies. We ignored HG002 when counting alleles because we will use this sample for benchmarking later.

We merged the common polymorphic LCRs and GRCh38 LCRs and added 5bp to both ends of each LCR because LCR boundaries may not be exact and by convention, insertions are often placed right before exact tandem repeats. This resulted in a BED file with 111,067 records, covering 35.4Mb of GRCh38. 29,291 records overlap with common polymorphic LCRs in the HPRC minigraph graph. 3,918 of them are not observed on GRCh38. 16.2% of the LCRs are intersected with the SegDup annotation from the "genomicSuperDups" track of the UCSC Genome Browser [11]. We see the overlap because an LCR consisting of several copies of a long repeat unit could also be considered as a SegDup.

LCRs are closely related to tandem repeats. Longdust identifies most tandem repeats of ≥50bp with ≥4 exact copies of repeat units as LCRs [8], though it often misses tandem repeats with fewer copies and may report additional regions without clear tandem patterns. 83.5% of our LCRs overlap with tandem repeats found by TRF [12] v4.10 (option `2 7 7 80 10 50 500 -l12`) and 92.0% overlap with TR Catalog [13] v1.2.1. This catalog covers 238Mb of GRCh38, much larger than our regions.

We applied the same procedure to the T2T-CHM13 genome [14] and found 79.6Mb of LCRs, doubling the length of LCRs in GRCh38. Most of the additional regions came from centromeric satellites that are not HSAT2/3 or alpha repeats. If we exclude all types of satellites [15], only 31.2Mb is left. The remaining difference in size from GRCh38 LCRs is probably caused by satellite annotation.

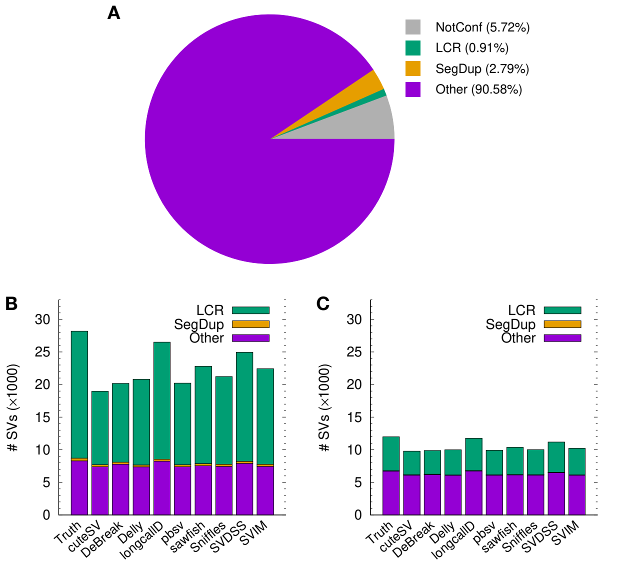

Figure 1. Number of HG002 structural variants (SVs) on GRCh38. (A) Lengths of regions. "NotConf" denotes not-confident regions in the HG002-Q100 v1.1 benchmark, excluding assembly gaps in GRCh38. A region classified to a previous type will not be counted towards the next type in the order of NotConf, LCR (low-complexity region), SegDup (segmental duplication) and Other. (B) Number of HG002-Q100 SVs stratified by LCR, SegDup and the rest of the confident regions. An SV is classified as LCR (or SegDup) if ≥70% of its interval on GRCh38 overlaps with LCR (or SegDup). An SV classified as LCR will not be classified as SegDup. (C) Number of HG002-Q100 SVs in the HG002-SV v0.6 confident regions lifted over from GRCh37.

Data Analysis

To understand the effect of LCRs in long-read SV calling, we measured the accuracy of SV calls stratified by LCR. We called SVs with 11 callers and compared them to both the new HG002-Q100 v1.1 [4] and the old HG002-SV v0.6 [5] benchmarks to demonstrate the impact of LCR in SV calling.

Investigating the GIAB truth SVs

There are 29,131 SVs of ≥50bp in length contained in the confident regions in the new HG002-Q100 v1.1 benchmark [4]. 943 of them have "*" as alternate alleles. We manually inspected the read alignment around some of these SVs and believe they are all redundant. Removing them from the truth left us with 28,188 SVs. The truvari [16] evaluation tool also filters SVs with "*" alleles.

The older HG002-SV v0.6 benchmark [5] is only available in the GRCh37 coordinate. To evaluate the SV calling accuracy on this benchmark, we lifted its confident regions over to GRCh38 with UCSC's liftover web service which failed on 0.03% of intervals. We still took SVs from HG002-Q100 as the ground truth. There are 11,985 HG002-Q100 overlapping with the lifted HG002-SV confident regions, more than the 9,705 SVs from the older HG002-SV benchmark. The difference is caused by the allele resolution. Suppose both haplotypes in HG002 harbor a 6kb insertion to the same location of the reference genome. The inserted sequences however differ by one SNP between them. The newer HG002-Q100 benchmark would consider this event as two heterozygous insertions, but the older HG002-SV benchmark would merge the two insertion alleles and consider them as one homozygous insertion. As a result, we counted 7,362 insertions in HG002-Q100 v1.1 but only 5,444 in HG002-SV v0.6, a sharp reduction. At the same time, the allele resolution may also affect deletions. If there are overlapping deletions of similar lengths between the two haplotypes, HG002-Q100 will encode them two independent deletions, but HG002-SV may merge them and thus reduce the total counts. Overall, constructed from long-read assemblies, HG002-Q100 is more precise and more accurate than HG002-SV.

Calling SVs from long reads

We acquired PacBio High-Fidelity reads from HPRC [17], aligned them to the primary assembly of GRCh38 with minimap2 [18] and called SVs with cuteSV v2.1.1 [19], DeBreak v1.0.2 [20], Delly v1.3.3 [21], longcallD v0.0.5 [22], pbsv v2.11.0 [23], Sawfish v0.12.10 [24], Sniffles2 v2.6.3 [6], SVDSS v2.1.0 [25], and SVIM v2.0.0 [26]. We used kanpig v1.1.0 [13] for genotyping SVs called by SVDSS as is suggested in the documentation. Sniffles2 may optionally take tandem repetitive regions as input, but using this option slightly reduced its overall accuracy, so we only evaluated its default setting.

We also tried specialized tandem repeat callers including TRGT [27] and ATaRVa [28]. These tools may output both long reference and long alternate alleles for one variant. Truvari was unable to correctly evaluate such variants and greatly overestimated false positives. We thus did not include tandem repeat callers in this work.

Most SVs are located in LCRs

We stratified HG002 SVs by LCR and SegDup (Fig. 1). For an SV to be classified as LCR or SegDup, we required it to have large overlap with LCR or SegDup regions. Without this condition, a long deletion containing a short LCR would be falsely classified as LCR, which would inflate the number of LCR SVs. Across the SV callers, 59.4–67.7% of SV calls overlap with LCR, although LCR only accounts for 1.2% of GRCh38 or 0.9% of GIAB confident regions. SVs are highly enriched in LCR. In the ground truth, SVs in LCR contributed to 42.1% of total SV lengths. This suggests these SVs are shorter than the average.

Whereas the numbers of "Other" SVs in the HG002-Q100 confi-

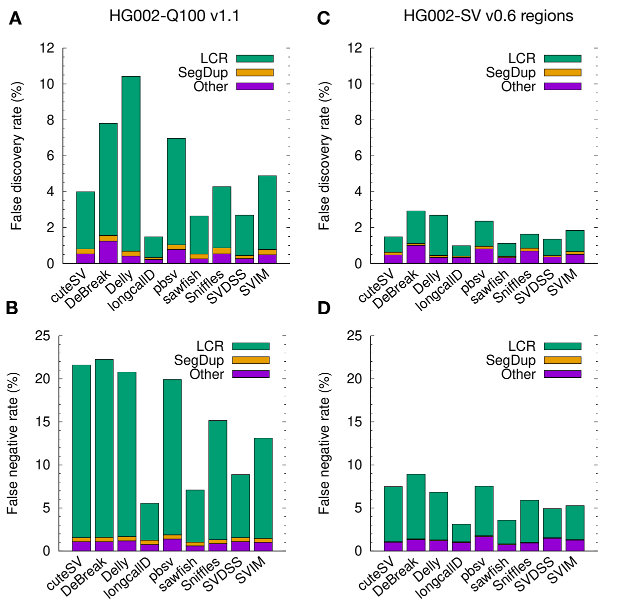

Figure 2. Accuracy of SV calls. (A) False discovery rate (FDR) of SVs in the HG002-Q100 confident regions, measured by truvari in the "refine" mode. SVs are stratified to LCR, SegDup and Other in the same way as is described in Fig. 1. (B) False negative rate (FNR) of SVs in HG002-Q100. (C) FDR in the HG002-SV confident regions. (D) FNR in HG002-SV.

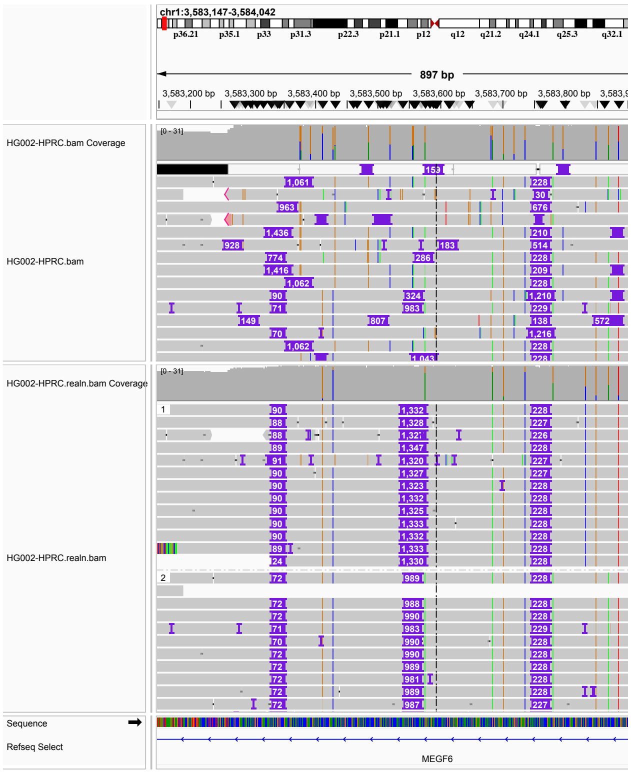

Figure 3. IGV screenshot of alignment around an LCR. The top panel shows the raw alignment by minimap2. The bottom panel shows the phased realignment by longcallD. There are 1650 (=90+1332+228) inserted bases on the first haplotype in total and 1290 (=72+990+228) inserted bases on the second haplotype, identical to the HG002-Q100 ground truth.

dent regions are similar across callers, the numbers of LCR SVs differ greatly (Fig. 1B). SV callers that attempt to produce haplotype-resolved SVs, such as longcallD and SVDSS, call noticeably more SVs in LCR and SegDup. This trend is also observed in the older HG002-SV v0.6 confident regions (Fig. 1C). In the older HG002-SV regions, there are much fewer SVs in LCR and almost none in SegDup, although the numbers of SVs in Other regions are only reduced a little. This indicates that the main difference between HG002-Q100 and HG002-SV comes from LCRs.

SVs in LCRs are harder to call correctly

We evaluated SV calls with truvari v5.3.0 [16], which performs multi-sequence alignment to normalize different variant representations and is recommended by GIAB. Having explored multiple truvari options, we settled on "`bench --passonly --pick ac --dup-to-ins`" followed by "`refine --use-original-vcfs`" as the resulting accuracy matched our manual inspection better.

On the new HG002-Q100 benchmark, 31.1–39.0% of SVs, depending on callers, are marked as "Other" (Fig. 1B), but only 5.2–14.0% of SV errors come from "Other" (Fig. 2A and 2B). This suggests SVs in the Other category are easier to call. In contrast, the majority of errors, at 77.3–91.3%, are located in LCRs. SVs in SegDup are also difficult to call, but due to the small number of such SVs, they do not contribute much to the total number of errors. Furthermore, 66% of truth SVs are insertions. The error rates of insertions and of deletions are similar across callers, mostly within a few percent.

Developed in our group but unpublished, longcallD achieves the lowest error rate (Fig. 2) mainly because it performs haplotype-aware multi-sequence realignment. As is shown in the top panel of Fig. 3, minimap2 often places gaps differently across reads and thus produces inconsistent alignment in long LCRs. This is primarily caused by the impure tandem repeat (TRF reports a 18bp repeat unit of 91% averaged identity) where a small sequencing error may greatly alter gap placement. Minimap2 would not produce the desired alignment because it does not see other reads in the same region during pairwise alignment. Such inconsistency would confuse most SV callers. For this example, the SV callers in the order shown in Fig. 2, respectively, called +1007/+1007, +1392/+1392, +1293/+1293, +1650/+1290, +1278/+1278, +1278/+1668, +963/+1191, +1650/+1290 and +1353/+2306 insertions on the two haplotypes. Only longcallD and SVDSS found the precise allele lengths of +1650/+1290. Nonetheless, truvari considered all callers correct. The error rate of most callers would probably be higher if we required precise allele matches.

We further stratified the errors by the maximum allele length of each LCR (Fig. 4) and observed increased error rates with maximum allele lengths. Some callers missed about half of SVs in ≥2kb LCRs, even though HiFi reads are long enough to span most them. Simple algorithms without realignment or reassembly are not capable of calling SVs in long LCRs.

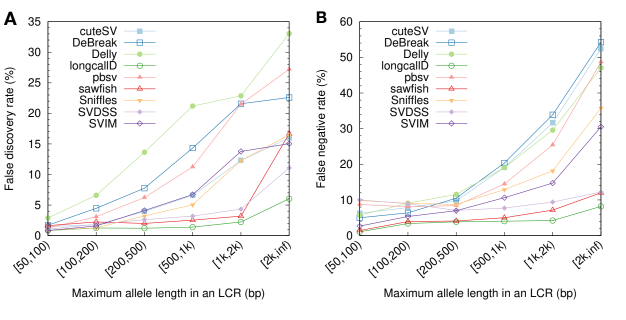

Figure 4. Accuracy of SV calls stratified by the maximum allele length in LCR. If an LCR is a common polymorphism (supported by ≥5 non-GRCh38 assemblies in HPRC), the maximum allele length equals to the length of the longest allele aligned to the LCR; otherwise, the maximum allele length equals to the length of the LCR on GRCh38.

Discussion

LCR SVs are a distinct class. Although LCRs only contribute to 1.2% of GRCh38 excluding alpha and HSAT2/3 repeats, they harbor more than half of long-read SV calls and an even higher fraction of SV calling errors. These errors are mainly caused by inconsistent read alignment especially around long LCRs. Short-read SV calling may be affected more due to uncertainty in alignment around LCRs. On the other hand, we note that LCRs may overlap with coding exons of genes that have functional impacts [29] and they may also mediate gene expression [30, 31]. We would not want to filter all SVs overlapping LCRs.

For data analysts, we recommend stratifying SVs by LCR as LCR SVs are enriched with errors and are resulted from different biological processes. For developers, we would like to emphasize the critical role of realignment or local reassembly in accurate SV calling. Most SVs in LCRs can still be called to decent accuracy with good algorithms.

Given accurate long reads at high coverage, we may also assemble the reads with haplotype-resolved assemblers [32, 33] and call variants from assembly-to-reference alignment [34]. Performing phasing and alignment within each haplotype, these assemblers are more powerful than most SV callers. As a matter of fact, the HG002-Q100 truth was derived this way.

We have only analyzed one human sample in this article. If mainstream SV callers are already struggling with long LCRs, merging their calls across different samples will be more problematic. When haplotype-resolved assembly is possible, calling variants across samples with pangenome-based methods [10, 35, 36] will be the preferred approach as conducting multi-sequence alignment across samples, such methods can produce more consistent SV representations. They may also struggle with highly variable LCRs, but will do better than traditional SV merging in most cases.

Data Availability

Low-complexity regions (LCRs) are available at Zenodo [37] (file "`chm13v2.lcr-v4.bed.gz`" and "`hg38.lcr-v4.bed.gz`"). Scripts used for producing the LCRs and plots can be found at GitHub [38].

Declarations

List of abbreviations

GIAB: Genome-In-A-Bottle group; HPRC: Human Pangenome Reference Consortium; kb: kilobase; LCR: low-complexity regions; Mb: megabase; SegDup: segmental duplication; SV: structural variant.

Competing Interests

The authors declare they have no competing interests.

Funding

This work is supported by National Institute of Health grant R01HG010040, R01HG014175, U24CA294203, U01HG013748 and U41HG010972 (to H.L.).

Author's Contributions

H.L. conceived the project. Q.Q. produced structural variant calls. Q.Q. and H.L. analyzed the data and drafted the manuscript.

Acknowledgements

We would like to acknowledge the National Genome Research Institute (NHGRI) for funding the following grants supporting the creation of the human pangenome reference: U41HG010972, U01HG010971, U01HG013760, U01HG013755, U01HG013748, U01HG013744, R01HG011274, and the Human Pangenome Reference Consortium (BioProject ID: PRJNA730823).

References

1. Eichler EE. Genetic Variation, Comparative Genomics, and the Diagnosis of Disease. N Engl J Med 2019;381:64–74.
2. Liao WW, Asri M, Ebler J, Doerr D, Haukness M, et al. A draft human pangenome reference. Nature 2023;617:312–324.
3. Logsdon GA, Ebert P, Audano PA, Loftus M, Porubsky D, et al. Complex genetic variation in nearly complete human genomes. Nature 2025;644:430–441.
4. Hansen NF, Dwarshuis N, Ji HJ, Rhie A, Loucks H, et al. A complete diploid human genome benchmark for personalized genomics. bioRxiv 2025;p. 2025.09.21.677443.
5. Zook JM, Hansen NF, Olson ND, Chapman L, Mullikin JC, et al. A robust benchmark for detection of germline large deletions and insertions. Nat Biotechnol 2020;38:1347–1355.
6. Smolka M, Paulin LF, Grochowski CM, Horner DW, Mahmoud M, et al. Detection of mosaic and population-level structural variants with Sniffles2. Nat Biotechnol 2024;42:1571–1580.
7. Keskus AG, Bryant A, Ahmad T, Yoo B, Aganezov S, et al. Severus detects somatic structural variation and complex rearrangements in cancer genomes using long-read sequencing. Nat Biotechnol 2025;.
8. Li H, Li B. Finding low-complexity DNA sequences with longdust. ArXiv 2025;p. arXiv:2509.07357v1.
9. Li H. Identifying centromeric satellites with dna-brnn. Bioinformatics 2019;35:4408–4410.
10. Li H, Feng X, Chu C. The design and construction of reference pangenome graphs with minigraph. Genome Biol 2020;21:265.
11. Perez G, Barber GP, Benet-Pages A, Casper J, Clawson H, et al. The UCSC Genome Browser database: 2025 update. Nucleic Acids Res 2025;53:D1243–D1249.
12. Benson G. Tandem repeats finder: a program to analyze DNA sequences. Nucleic Acids Res 1999;27:573–80.
13. English AC, Cunial F, Metcalf GA, Gibbs RA, Sedlazeck FJ. K-mer analysis of long-read alignment pileups for structural variant genotyping. Nat Commun 2025;16:3218.
14. Nurk S, Koren S, Rhie A, Rautiainen M, Bzikadze AV, et al. The complete sequence of a human genome. Science 2022;376:44–53.
15. Altemose N, Logsdon GA, Bzikadze AV, Sidhwani P, Langley SA, et al. Complete genomic and epigenetic maps of human centromeres. Science 2022;376:eabl4178.
16. English AC, Menon VK, Gibbs RA, Metcalf GA, Sedlazeck FJ. Truvari: refined structural variant comparison preserves allelic diversity. Genome Biol 2022;23:271.
17. Human Pangenome Reference Consortium;. https://human-pangenomics.s3.amazonaws.com/submissions/80d00e88-7a92-46d8-88c7-48f1486e11ed--HG002_PACBIO_REVIO/m84039_230117_233243_s1.hifi_reads.default.bam.
18. Li H. Minimap2: pairwise alignment for nucleotide sequences. Bioinformatics 2018;34:3094–3100.
19. Jiang T, Liu Y, Jiang Y, Li J, Gao Y, et al. Long-read-based human genomic structural variation detection with cuteSV. Genome Biol 2020;21:189.
20. Chen Y, Wang AY, Barkley CA, Zhang Y, Zhao X, et al. Deciphering the exact breakpoints of structural variations using long sequencing reads with DeBreak. Nat Commun 2023;14:283.
21. Rausch T, Zichner T, Schlattl A, Stütz AM, Benes V, Korbel JO. DELLY: structural variant discovery by integrated paired-end

and split-read analysis. Bioinformatics 2012;28:i333–i339.
22. ;. https://github.com/yangao07/longcallD.
23. ;. https://github.com/PacificBiosciences/pbsv.
24. Saunders CT, Holt JM, Baker DN, Lake JA, Belyeu JR, et al. Sawfish: improving long-read structural variant discovery and genotyping with local haplotype modeling. Bioinformatics 2025;41:btaf136.
25. Denti L, Khorsand P, Bonizzoni P, Hormozdiari F, Chikhi R. SVDSS: structural variation discovery in hard-to-call genomic regions using sample-specific strings from accurate long reads. Nat Methods 2023;20:550–558.
26. Heller D, Vingron M. SVIM: structural variant identification using mapped long reads. Bioinformatics 2019;35:2907–2915.
27. Dolzhenko E, English A, Dashnow H, De Sena Brandine G, Mokveld T, et al. Characterization and visualization of tandem repeats at genome scale. Nat Biotechnol 2024;42:1606–1614.
28. Sivakumar AK, Sudarsanam S, Sharma A, Avvaru AK, Sowpati DT. ATaRVa: Analysis of Tandem Repeat Variation from Long Read Sequencing data. bioRxiv 2025;p. 2025.05.13.653434.
29. Mukamel RE, Handsaker RE, Sherman MA, Barton AR, Zheng Y, et al. Protein-coding repeat polymorphisms strongly shape diverse human phenotypes. Science 2021;373:1499–1505.
30. Bakhtiari M, Park J, Ding YC, Shleizer-Burko S, Neuhausen SL, et al. Variable number tandem repeats mediate the expression of proximal genes. Nat Commun 2021;12:2075.
31. Lu TY, Smaruj PN, Fudenberg G, Mancuso N, Chaisson MJP. The motif composition of variable number tandem repeats impacts gene expression. Genome Res 2023;33:511–524.
32. Cheng H, Concepcion GT, Feng X, Zhang H, Li H. Haplotype-resolved de novo assembly using phased assembly graphs with hifiasm. Nat Methods 2021;18:170–175.
33. Rautiainen M, Nurk S, Walenz BP, Logsdon GA, Porubsky D, et al. Telomere-to-telomere assembly of diploid chromosomes with Verkko. Nat Biotechnol 2023;41:1474–1482.
34. Li H, Bloom JM, Farjoun Y, Fleharty M, Gauthier L, et al. A synthetic-diploid benchmark for accurate variant-calling evaluation. Nat Methods 2018;15:595–597.
35. Hickey G, Monlong J, Ebler J, Novak AM, Eizenga JM, et al. Pangenome graph construction from genome alignments with Minigraph-Cactus. Nat Biotechnol 2024;42:663–673.
36. Garrison E, Guarracino A, Heumos S, Villani F, Bao Z, et al. Building pangenome graphs. Nat Methods 2024;21:2008–2012.
37. ;. https://doi.org/10.5281/zenodo.10903864.
38. ;. https://github.com/lh3/lcr-sv.

Manuscript
Click here to access/download;Manuscript;lcr-sv.pdf

Your PDF file "lcr-sv.pdf" cannot be opened and processed. Please see the common list of problems, and suggested resolutions below.

Reason:

Other Common Problems When Creating a PDF from a PDF file

You will need to convert your PDF file to another format or fix the current PDF file, then re-submit it.

Reviewer #1

Q2.1.1: My primary concern from the initial review remains insufficiently addressed. The manuscript continues to rebrand tandem repeats as low complexity regions. This is problematic because low complexity region is a technical label produced by tools like long dust, not a widely accepted biological category. The authors note that 83 percent of these low complexity regions overlap with tandem repeats, which strongly suggests they are simply tandem repeats as detected by long dust.

This approach creates two issues. First, it overlooks the large body of existing tandem repeat research, much of which predates long read sequencing. Second, it makes the main message less clear. The core point is that tandem repeats are often detected by structural variant callers but are fragmented, miscalled, or misannotated in downstream analysis. Reviewer 3 raised a similar concern.

I have no concerns about the validity of the analysis, and I believe this work should be published in G4. Revisions can be made after review in G4, and the responsibility now lies with the authors to improve the clarity and flow of the manuscript and better place the work in the context of tandem repeat research.

They can contact me if further discussion would be useful, since G4 is open review.

A2.1.1: "Low complexity" is not a new concept coined by us. Andrey Kolmogorov defined Kolmogorov string complexity in 1963. BLASTn has been masking low-complexity sequences for over two decades. Longdust is built upon SDUST, an algorithm published in 2006 and currently used by BLASTn (BLASTn was using a less stringent version of DUST before 2006). Please see ref [8] for a complete literature review.

As we point out in the manuscript and in ref [8], tandem repeat and LCR have large overlaps. We are well aware of the literature on tandem repeats, but we prefer "low complexity" for two reasons. First, unlike "low complexity", impure tandem repeat lacks a mathematical definition. We cannot tell if a sequence is an impure tandem repeat without running a tool like TRF, TANTAN, ULTRA or Pytrf. These tools report noticeably different results [PMID: 40462000 and ref 8]. This is partly why tandem repeat catalogs vary greatly. We are not comfortable with this level of variability. Second, TRF, the most widely used tool for finding tandem repeats, is >20 times slower than longdust and uses >15 times more memory. We have applied longdust to all 462 HPRC genomes to identify LCRs expanded beyond the reference genome. It is cumbersome to use TRF due to its inefficiency.

Furthermore, we note that pure tandem repeats would not cause alignment inconsistencies; impure tandem repeats are the culprit. In the revision, we provide more explanation to the effect of impure tandem repeats on minimap2 alignment: "*As is shown in the top panel of Fig. 3, minimap2 often places gaps differently across reads and thus produces inconsistent alignment in long LCRs. This is primarily caused by impure tandem repeat (TRF reports a 18bp repeat unit of 91% averaged identity in the middle) where a small sequencing error may greatly alter gap*

placement. Minimap2 would not produce the desired alignment because it does not see other reads in the same region during pairwise alignment."

Reviewer #3

Q2.3.1: Comment 1 on A1.3.1
The statement "LCR is closely related to tandem repeat sequences" is too brief as an explanation of LCR. Please provide a more detailed explanation of the difference between LCR and tandem repeat sequences (TR) in the manuscript. Since the term LCR is more frequently used in the context of protein sequences, it may cause confusion for readers.

A2.3.1: We now comment that "*Longdust identifies most tandem repeats of ⩾50bp with ⩾4 exact copies of repeat units as LCRs, though it often misses tandem repeats with fewer copies and may report additional regions without clear tandem patterns.*" We note that "low complexity" is not specific to protein sequences. BLASTn has been masking low-complexity nucleotide sequences for over two decades. Please see ref [8] for a complete literature review.

Q2.3.2: Comment 2 on A1.3.2
If the type of error cannot be determined by Travari, the authors provide FDR and FNR for each SV type (i.e., INS and DEL). Information on which SV type causes more errors in LCR would be beneficial to readers.

A2.3.2: We added that "*66% of truth SVs are insertions. The error rates of insertions and of deletions are similar across callers, mostly within a few percent.*"
