# Supplementary material for: Challenges in structural variant calling in low-complexity regions
Source: Gigascience. 2025 Dec 12;14:giaf154. doi: 10.1093/gigascience/giaf154 (PMC12758381; doi:10.1093/gigascience/giaf154)
Supplement: giaf154_Reviewer_1_Report_Original_Submission — Zev Kronenberg, Ph.D. -- 10/21/2025 [file giaf154_reviewer_1_report_original_submission.pdf]

Reviewer Report

Title: Challenges in structural variant calling in low-complexity regions

Version: Original Submission **Date:** 10/21/2025

Reviewer name: Zev Kronenberg, Ph.D.

Reviewer Comments to Author:

Summary StatementThis manuscript addresses an important and timely topic in genome analysis, and the authors' effort to systematically examine low-complexity regions is commendable. The study has the potential to contribute valuable insights into the challenges of variant interpretation in difficult genomic contexts. However, several critical analyses are missing, and the presentation would benefit from significant reorganization and clarification. In particular, the relationship between tandem repeats and low-complexity regions is not sufficiently explored, which limits the interpretability and completeness of the findings. With additional analyses and improved focus, this work could make a meaningful contribution to the field.Major Comments1. Incomplete treatment of tandem repeats (TRs)The manuscript does not sufficiently address the overlap between low-complexity sequences and tandem repeats (TRs). TRs often coincide with low-complexity regions, small indel variants, and structural variation, making them an important genomic feature to consider. Without incorporating TR context, the analyses risk being oversimplified. For example, in Figure 3, the depicted region corresponds to an entry in the Adotto tandem repeat catalog (chr1:3583240-3583962; https://github.com/ACEnglish/adotto). The authors should clarify how such regions were handled and whether TRs were explicitly excluded, annotated, or analyzed separately.2. Performance of TR/VNTR callers in low-complexity regionsA natural extension of the above point concerns how tandem repeat or VNTR-specific tools (for example, TRGT [Dolzhenko], Vamos [Chaisson], and others) would perform within these "LCRs." Many of these regions are, in fact, tandem repeats, and it would strengthen the manuscript to discuss or benchmark the performance of these specialized tools in that context. Even a qualitative discussion would add depth and help position the current work within the broader methodological landscape.3. Quantification of variation by sequence lengthThe manuscript primarily reports counts of variants, particularly structural variants, without contextualizing them by the amount of sequence affected. Given that a typical human genome differs from the reference by roughly 20 Mb of structural variation, it would be more informative to describe results in terms of sequence length impacted rather than event counts alone. This approach would better reflect the biological impact and improve interpretability of the results.4. Clarity and structure of the proseThe writing would benefit from careful editing to improve clarity and logical flow. At present, it can be difficult to identify the central findings and their supporting evidence. The authors are encouraged to restructure the manuscript to guide the reader more clearly through the rationale, methods, results, and conclusions.Specific examples include:o "This article gives the answer" is conversational and not typical for scientific literature.o "We will also explain caveats in the truth data" has an unclear meaning and seems incomplete.o "Developed in our group" should be revised, as LongCallD does not appear to be a

published or preprinted method. To avoid confusion, the authors might clarify the status of the tool or use more neutral phrasing.

Level of Interest

Please indicate how interesting you found the manuscript: Choose an item.

Quality of Written English

Please indicate the quality of language in the manuscript: Choose an item.

Declaration of Competing Interests

Please complete a declaration of competing interests, considering the following questions:

- Have you in the past five years received reimbursements, fees, funding, or salary from an organisation that may in any way gain or lose financially from the publication of this manuscript, either now or in the future?
- Do you hold any stocks or shares in an organisation that may in any way gain or lose financially from the publication of this manuscript, either now or in the future?
- Do you hold or are you currently applying for any patents relating to the content of the manuscript?
- Have you received reimbursements, fees, funding, or salary from an organization that holds or has applied for patents relating to the content of the manuscript?
- Do you have any other financial competing interests?
- Do you have any non-financial competing interests in relation to this paper?

If you can answer no to all of the above, write 'I declare that I have no competing interests' below. If your reply is yes to any, please give details below.

I have no direct competing interests. I do wish to declare I am an employee and shareholder in PacBio, and a shareholder of Phase Genomics.

I agree to the open peer review policy of the journal. I understand that my name will be included on my report to the authors and, if the manuscript is accepted for publication, my named report including any attachments I upload will be posted on the website along with the authors' responses. I agree for my report to be made available under an Open Access Creative Commons CC-BY license (http://creativecommons.org/licenses/by/4.0/). I understand that any comments which I do not wish to be included in my named report can be included as confidential comments to the editors, which will not be published.

Choose an item.

To further support our reviewers, we have joined with Publons, where you can gain additional credit to further highlight your hard work (see: https://publons.com/journal/530/gigascience). On publication of this paper, your review will be automatically added to Publons, you can then choose whether or not to claim your Publons credit. I understand this statement.

Yes Choose an item.
