# Supplementary material for: Challenges in structural variant calling in low-complexity regions
Source: Gigascience. 2025 Dec 12;14:giaf154. doi: 10.1093/gigascience/giaf154 (PMC12758381; doi:10.1093/gigascience/giaf154)
Supplement: giaf154_Reviewer_1_Report_Revision_1 — Zev Kronenberg, Ph.D. -- 12/4/2025 [file giaf154_reviewer_1_report_revision_1.pdf]

Reviewer Report

Title: Challenges in structural variant calling in low-complexity regions

Version: Revision 1 **Date:** 12/4/2025

Reviewer name: Zev Kronenberg, Ph.D.

Reviewer Comments to Author:

My primary concern from the initial review remains insufficiently addressed. The manuscript continues to rebrand tandem repeats as low complexity regions. This is problematic because low complexity region is a technical label produced by tools like long dust, not a widely accepted biological category. The authors note that 83 percent of these low complexity regions overlap with tandem repeats, which strongly suggests they are simply tandem repeats as detected by long dust.This approach creates two issues. First, it overlooks the large body of existing tandem repeat research, much of which predates long read sequencing. Second, it makes the main message less clear. The core point is that tandem repeats are often detected by structural variant callers but are fragmented, miscalled, or misannotated in downstream analysis. Reviewer 3 raised a similar concern.I have no concerns about the validity of the analysis, and I believe this work should be published in G4. Revisions can be made after review in G4, and the responsibility now lies with the authors to improve the clarity and flow of the manuscript and better place the work in the context of tandem repeat research.They can contact me if further discussion would be useful, since G4 is open review.

Level of Interest

Please indicate how interesting you found the manuscript: Choose an item.

Quality of Written English

Please indicate the quality of language in the manuscript: Choose an item.

Declaration of Competing Interests

Please complete a declaration of competing interests, considering the following questions:

- Have you in the past five years received reimbursements, fees, funding, or salary from an organisation that may in any way gain or lose financially from the publication of this manuscript, either now or in the future?
- Do you hold any stocks or shares in an organisation that may in any way gain or lose financially from the publication of this manuscript, either now or in the future?
- Do you hold or are you currently applying for any patents relating to the content of the manuscript?

- Have you received reimbursements, fees, funding, or salary from an organization that holds or has applied for patents relating to the content of the manuscript?
- Do you have any other financial competing interests?
- Do you have any non-financial competing interests in relation to this paper?

If you can answer no to all of the above, write 'I declare that I have no competing interests' below. If your reply is yes to any, please give details below.

I am an employee and shareholder of PacBio. I am a share holder in Phase Genomics. I have no direct conflicts of interest.

I agree to the open peer review policy of the journal. I understand that my name will be included on my report to the authors and, if the manuscript is accepted for publication, my named report including any attachments I upload will be posted on the website along with the authors' responses. I agree for my report to be made available under an Open Access Creative Commons CC-BY license (http://creativecommons.org/licenses/by/4.0/). I understand that any comments which I do not wish to be included in my named report can be included as confidential comments to the editors, which will not be published.

Choose an item.

To further support our reviewers, we have joined with Publons, where you can gain additional credit to further highlight your hard work (see: https://publons.com/journal/530/gigascience). On publication of this paper, your review will be automatically added to Publons, you can then choose whether or not to claim your Publons credit. I understand this statement.

Yes Choose an item.
