# Supplementary material for: Challenges in structural variant calling in low-complexity regions
Source: Gigascience. 2025 Dec 12;14:giaf154. doi: 10.1093/gigascience/giaf154 (PMC12758381; doi:10.1093/gigascience/giaf154)
Supplement: giaf154_Revision_2_Report_Original_Submission — Wan-Ping Lee -- 10/31/2025 [file giaf154_revision_2_report_original_submission.pdf]

Reviewer Report

Title: Challenges in structural variant calling in low-complexity regions

Version: Original Submission **Date:** 10/31/2025

Reviewer name: Wan-Ping Lee

Reviewer Comments to Author:

The authors delineate low-complexity regions (LCRs) and show that although LCRs comprise only ~1.2% of GRCh38, they harbor the majority of confident SVs and the vast majority of calling errors across long-read callers; error rates rise with LCR length.Major comments1. You report 79.6 Mb of LCRs on T2T-CHM13 that drop to 31.2 Mb after excluding satellites, versus 35.4 Mb on GRCh38. If T2T-CHM13 truly "resolves" specific GRCh38 LCRs, please add 1-2 concrete locus examples to illustrate what is gained.2. Please detail the GRCh37â†'GRCh38 liftover for the v0.6 regions. Did you lift over entire intervals or only start and end coordinates? Also, how many SVs (and what proportion) failed liftover?3. Do you expect the conclusions to hold if reads are aligned to T2T-CHM13 instead of GRCh38?4. Because most public biobank data are short-read, LCRs are likely to exacerbate ambiguity, so both FNR and FDR may be higher than with long reads. Please add a brief note in the Discussion to set expectations and, if feasible, a small comparative run on HG002 using a standard short-read SV pipeline to show whether the LCR error gradient is amplified.Minor comment5. Abstract/Conclusion: "Special care need to be taken …" â†' "Special care needs to be taken …".

Level of Interest

Please indicate how interesting you found the manuscript: Choose an item.

Quality of Written English

Please indicate the quality of language in the manuscript: Choose an item.

Declaration of Competing Interests

Please complete a declaration of competing interests, considering the following questions:

- Have you in the past five years received reimbursements, fees, funding, or salary from an organisation that may in any way gain or lose financially from the publication of this manuscript, either now or in the future?
- Do you hold any stocks or shares in an organisation that may in any way gain or lose financially from the publication of this manuscript, either now or in the future?
- Do you hold or are you currently applying for any patents relating to the content of the manuscript?

- Have you received reimbursements, fees, funding, or salary from an organization that holds or has applied for patents relating to the content of the manuscript?
- Do you have any other financial competing interests?
- Do you have any non-financial competing interests in relation to this paper?

If you can answer no to all of the above, write 'I declare that I have no competing interests' below. If your reply is yes to any, please give details below.

I declare that I have no competing interests.

I agree to the open peer review policy of the journal. I understand that my name will be included on my report to the authors and, if the manuscript is accepted for publication, my named report including any attachments I upload will be posted on the website along with the authors' responses. I agree for my report to be made available under an Open Access Creative Commons CC-BY license (http://creativecommons.org/licenses/by/4.0/). I understand that any comments which I do not wish to be included in my named report can be included as confidential comments to the editors, which will not be published.

Choose an item.

To further support our reviewers, we have joined with Publons, where you can gain additional credit to further highlight your hard work (see: https://publons.com/journal/530/gigascience). On publication of this paper, your review will be automatically added to Publons, you can then choose whether or not to claim your Publons credit. I understand this statement.

Yes Choose an item.
