# Supplementary material for: Challenges in structural variant calling in low-complexity regions
Source: Gigascience. 2025 Dec 12;14:giaf154. doi: 10.1093/gigascience/giaf154 (PMC12758381; doi:10.1093/gigascience/giaf154)
Supplement: giaf154_Revision_3_Report_Original_Submission — Shunichi Kosugi -- 11/6/2025 [file giaf154_revision_3_report_original_submission.pdf]

Reviewer Report

Title: Challenges in structural variant calling in low-complexity regions

Version: Original Submission **Date:** 11/6/2025

Reviewer name: Shunichi Kosugi

Reviewer Comments to Author:

This study reports a high error calling rate for structural variations (SVs) detected with long read data in low complexity regions (LCRs). The representation of the results appears valid, but the information regarding the accuracy of SV calling is insufficient.Major points:1. This study focused on analyzing SV calling accuracy in LCRs, defined as regions that harbor repeatedly occurring motifs. The authors should provide a more detailed explanation of LCR: clarifying whether LCRs include tandem repeats (TRs) such as short tandem repeats (STRs) and variable number of tandem repeats (VNTRs). If there are differences from general TR, they should describe those differences. If LCRs defined in this study do not include inverted repeats, the term TR may be more appropriate than LCR.The LCRs used in this study were identified using the logdust tool developed by the authors. It is necessary to explain the differences between TRs identified using TRF (e.g., a simpleRepeat.txt file available on the UCSC site) and LCRs.2. This study presents false discovery rate (FDR) and false negative rate (FNR) for SV calling (Figs. 2 and 4). However, it does not provide information on the type of errors causing high FDR and FNR in LCRs (e.g., errors in SV length or breakpoint distance between SV calls and reference SVs). Given that this study focuses on SV detection accuracy in LCRs, this information is essential.3. Within the TR region, which constitutes primary components of LCRs, insertions and deletions composed of the TR repeat units can be assigned to any position within the TR region. For example, in a 200 bp TR region consisting of 20 copies of a 10 bp repeat unit, a 100 bp insertion consisting of 10 copies of the same repeat unit could be potentially aligned at the beginning, middle, or end of the TR region in a long read or assembly alignment. In addition, as shown in Fig. 3 and a previous report (Genome Biol 26, 246 2025), an insertion sequence composed of identical or similar repeat units could be fragmented into multiple insertion sequences within a TR region in a long read or assembly alignment. Therefore, SVs may be assigned to different positions within an LCR depending on alignment tools, alignment parameters, and read/assembly data. That is true for the HG002-Q100 truth SV data, which is derived from the alignments of haplotype-resolved assemblies to GRCh38. The results in Fig. 4 likely reflect this effect. The use of TR genotyping tools, such as LongTR and TRGT, may resolve this issue. These points should be discussed in the manuscript.Minor point:In line 8 on page 2, 'added 5bp to both ends of each LCR': What is the rationale for adding 5 bp to both ends of each LCR?

Level of Interest

Please indicate how interesting you found the manuscript: Choose an item.

Quality of Written English

Please indicate the quality of language in the manuscript: Choose an item.

Declaration of Competing Interests

Please complete a declaration of competing interests, considering the following questions:

- Have you in the past five years received reimbursements, fees, funding, or salary from an organisation that may in any way gain or lose financially from the publication of this manuscript, either now or in the future?
- Do you hold any stocks or shares in an organisation that may in any way gain or lose financially from the publication of this manuscript, either now or in the future?
- Do you hold or are you currently applying for any patents relating to the content of the manuscript?
- Have you received reimbursements, fees, funding, or salary from an organization that holds or has applied for patents relating to the content of the manuscript?
- Do you have any other financial competing interests?
- Do you have any non-financial competing interests in relation to this paper?

If you can answer no to all of the above, write 'I declare that I have no competing interests' below. If your reply is yes to any, please give details below.

I declare that I have no competing interests.

I agree to the open peer review policy of the journal. I understand that my name will be included on my report to the authors and, if the manuscript is accepted for publication, my named report including any attachments I upload will be posted on the website along with the authors' responses. I agree for my report to be made available under an Open Access Creative Commons CC-BY license (http://creativecommons.org/licenses/by/4.0/). I understand that any comments which I do not wish to be included in my named report can be included as confidential comments to the editors, which will not be published.

Choose an item.

To further support our reviewers, we have joined with Publons, where you can gain additional credit to further highlight your hard work (see: https://publons.com/journal/530/gigascience). On publication of this paper, your review will be automatically added to Publons, you can then choose whether or not to claim your Publons credit. I understand this statement.

Yes Choose an item.
